# Synergistic use of siderophores and weak organic ligands during zinc transport in the rhizosphere controlled by pH and ion strength gradients

**DOI:** 10.1038/s41598-022-10493-5

**Published:** 2022-04-26

**Authors:** George H. R. Northover, Yiru Mao, Salvador Blasco, Ramon Vilar, Enrique Garcia-España, Claudia Rocco, Md Hanif, Dominik J. Weiss

**Affiliations:** 1 Department of Earth Science and Engineering, Imperial College London, London, SW7 2AZ UK; 2 Instituto de Ciencia Molecular (ICMol), University of Valencia, 46980 Paterna, Spain; 3 Department of Chemistry, Imperial College London, London, W12 0BZ UK; 4 Department of Life Sciences, MRC Centre for Molecular Bacteriology and Infection, Imperial College London, London, UK; 5 Soil, Water and Environment Department, Khulna University, Khulna, Bangladesh; 6 Department of Civil and Environmental Engineering, Princeton University, Princeton, NJ 08540 USA

**Keywords:** Environmental chemistry, Element cycles, Element cycles

## Abstract

Citrate (Cit) and Deferoxamine B (DFOB) are two important organic ligands coexisting in soils with distinct different affinities for metal ions. It has been theorized that siderophores and weak organic ligands play a synergistic role during the transport of micronutrients in the rhizosphere, but the geochemical controls of this process remain unknown. Here we test the hypothesis that gradients in pH and ion strength regulate and enable the cooperation. To this end, first we use potentiometric titrations to identify the dominant Zn(II)–Cit and Zn(II)–DFOB complexes and to determine their ionic strength dependent stability constants between 0 and 1 mol dm^−3^. We parametrise the Extended Debye-Hückel (EDH) equation and determine accurate intrinsic association constants (logβ^0^) for the formation of the complexes present. The speciation model developed confirms the presence of [Zn(Cit)]^−^, [Zn(HCit)], [Zn_2_(Cit)_2_(OH)_2_]^4−^, and [Zn(Cit)_2_]^4−^, with [Zn(Cit)]^−^ and [Zn_2_(Cit)_2_(OH)_2_]^4−^ the dominant species in the pH range relevant to rhizosphere. We propose the existence of a new [Zn(Cit)(OH)_3_]^4−^ complex above pH 10. We also verify the existence of two hexadentate Zn(II)–DFOB species, i.e., [Zn(DFOB)]^−^ and [Zn(HDFOB)], and of one tetradentate species [Zn(H_2_DFOB)]^+^. Second, we identify the pH and ionic strength dependent ligand exchange points (LEP) of Zn with citrate and DFOB and the stability windows for Zn(II)–Cit and Zn(II)–DFOB complexes in NaCl and rice soil solutions. We find that the LEPs fall within the pH and ionic strength gradients expected in rhizospheres and that the stability windows for Zn(II)–citrate and Zn(II)–DFOB, i.e., low and high affinity ligands, can be distinctly set off. This suggests that pH and ion strength gradients allow for Zn(II) complexes with citrate and DFOB to dominate in different parts of the rhizosphere and this explains why mixtures of low and high affinity ligands increase leaching of micronutrients in soils. Speciation models of soil solutions using newly determined association constants demonstrate that the presence of dissolved organic matter and inorganic ligands (i.e., bicarbonate, phosphate, sulphate, or chlorides) do neither affect the position of the LEP nor the width of the stability windows significantly. In conclusion, we demonstrate that cooperative and synergistic ligand interaction between low and high affinity ligands is a valid mechanism for controlling zinc transport in the rhizosphere and possibly in other environmental reservoirs such as in the phycosphere. Multiple production of weak and strong ligands is therefore a valid strategy of plants and other soil organisms to improve access to micronutrients.

## Introduction

There is a diverse range of weak and strong binding organic ligands in the rhizosphere which originate from plants, bacteria, and fungi^1–3^. They are mainly secreted when there is a low supply of iron or other micronutrients in the environment^4^ and they often function as allelochemicals, i.e., a ligand released by one organism directly or indirectly influences the metal homeostasis of a neighbouring organism, be it of the same species or from a different taxonomic kingdom^5–7^. Acting as complexing ligands, they are associated with the cycling of a wide range of biologically important micronutrients in the rhizosphere^8–12^. The synergistic use of strong and weak organic ligands has been scarcely explored in the microenvironment of the rhizosphere due to experimental challenges associated with the small spatial scales^13^. This knowledge gap, however, undermines our understanding of how plants, bacteria and fungi acquire micronutrients from the soil environment and take them up. This in turn hinders attempts to improve fertilizer management and biofortification in rice and other crops or to understand the reason for the multiple siderophore production observed in soil organisms^2,14^.

Ligands forming weaker complexes with metal ions include monocarboxylic, dicarboxylic, or tricarboxylic ligands. Citrate is a tricarboxylic organic ligand, which is released by rice and soil-dwelling organisms and it is present in high concentrations in soils. Ligands forming strong complexes are siderophores which are composed of a limited set of metal-binding moieties: hydroxamate, catecholate and α-hydroxycarboxylate functionalities are the most common but α-aminocarboxylate and α-hydroxyimidazole functionalities have been reported as well^15,16^. They are found at much smaller concentrations. They are typically hexadentate ligands giving octahedral coordination geometries around the central metal^17^. Desferrioxamine B (DFOB) is a hydroxamic siderophore produced by the soil bacterium *Streptomyces pilosus* present abundantly in soils^18^.

**Figure 1:**
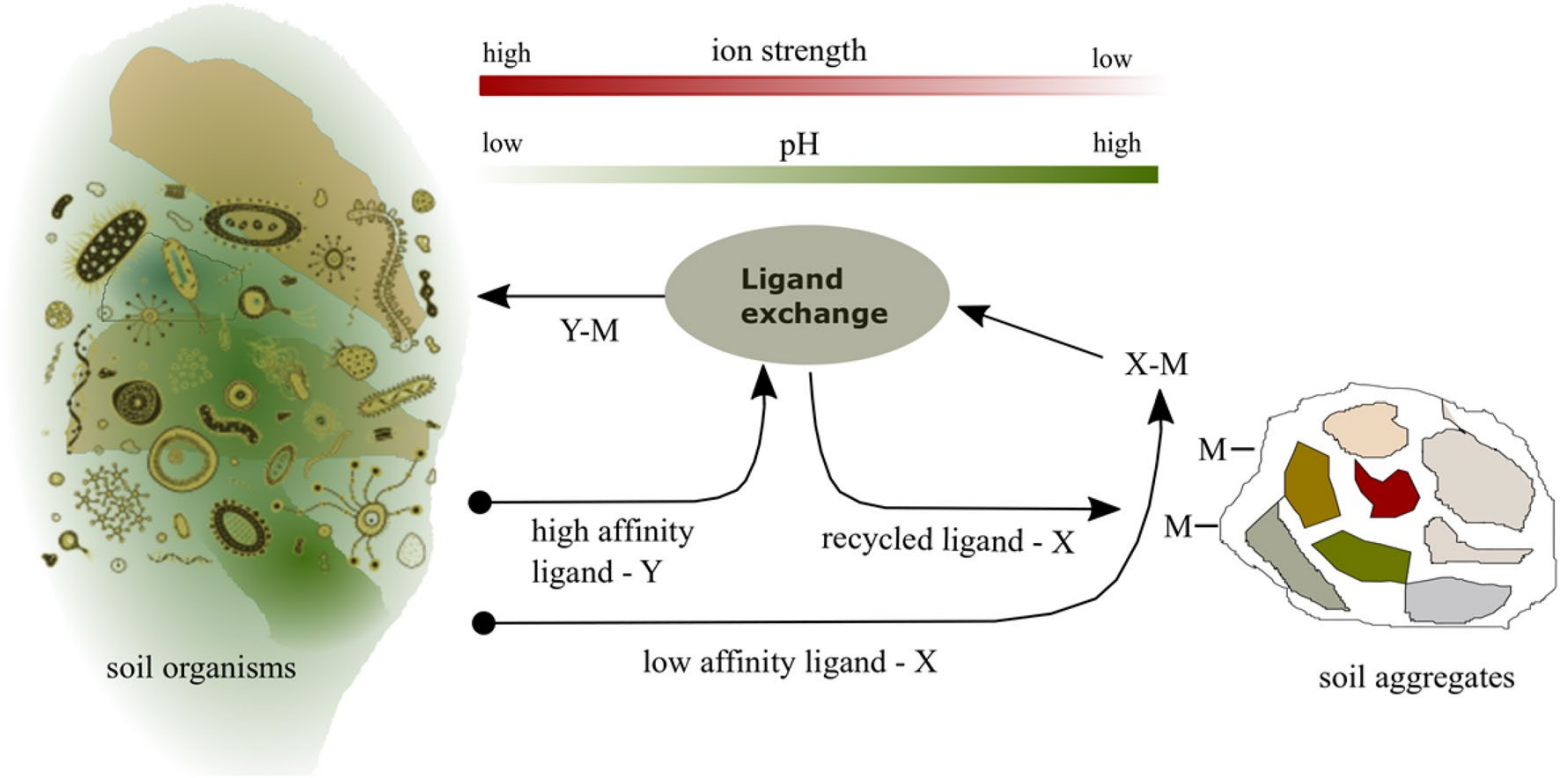
Conceptual model of possible interplay between two ligands (X,Y) with different binding affinities for metals (M). The model is drawn after the cartoon proposed by McRose and co-workers of high Fe affinity (Y) and low Fe affinity (X) siderophore interaction^2^. Formation of a M–X complex strips M from the soil aggregate and brings the metal into solution. The metal is then exchanged between X and Y, Y transports M towards cell surface whilst X is free to return to the soil aggregate. McRose identified X as a ligand with low affinity and Y as a ligand with high affinity. To confirm the possibility of a synergistic interaction between weak and strong organic ligands with different metal binding affinities during acquisition and transport in the rhizosphere, we need to identify the physicochemical conditions in the solution that enable the exchange of metal ions from one ligand to the other, the so-called ligand exchange point (LEP), and show that distinct different stabilty windows exist. Identifying stabilty windows is also crucial to answer the uncertainty if metal–ligand complexes are stable at the relevant interfaces in the rhizosphere, i.e., soil organisms (e.g., roots, bacteria, fungi) and soil aggregates (e.g., minerals, organic matter).

McRose and co-workers proposed a conceptual model for the possible interaction of different ligands released by bacteria during iron acquisition in natural solutions. This model is based upon previous mineral dissolution mechanism studies in the presence of both types of ligands^2,19,20^. The advantages of this pairing have been predicted by abiotic studies showing that a combination of siderophores and oxalate enhances Fe dissolution^19,20^. In it, as shown in Fig. 1, a ligand with low Fe affinity for example adsorbs to the mineral surface and strips the metal away from the mineral, bringing it into solution. After the formation of a labile complex at the mineral surface, a ligand exchange reaction takes place in which the metal is transferred to a ligand with high Fe affinity and the low affinity ligand is free to react with the mineral again. Modelling and experimental studies of organic ligand complexation with a wide range of first row transition metals have demonstrated that pH and ionic strength exert a critical influence on the stability and lability of metal–ligand complexes^3,21–24^. The rhizosphere indeed is characterised by microscale gradients in pH and ionic strength, so it is reasonable to expect that these two master variables play a central part in synergistic roles of siderophores and other organic ligands during mobilisation and transport of zinc in the rhizosphere.

Metal complexation to ligands in solution is fast, therefore thermodynamic speciation modelling can be used to determine metal speciation and explore geochemical mechanisms that cannot be studied experimentally^25^. Geochemical speciation modelling relies on knowing the concentrations of elements in the system, the species that form (i.e., the speciation model), and accurate stability constants for the relevant complexation reactions. Thermodynamic data always refer to a selected standard state and stability constants calculated at standard state (298.15 K, 1 atm, I = 0 mol dm^−3^), where concentrations are equal to activities with no ion interactions between dissolved species, are known as intrinsic stability constants ($\mathrm{log}{\upbeta }^{0}$). Stability constants measured under any other condition are conditional ($\mathrm{log }\upbeta$), their value depends on the chemical and physical environments (e.g., ionic strength, temperature, pressure) under which they are measured^26–28^. Two alternative methods are used to express the ionic medium dependence of equilibrium constants. One method takes into consideration the individual characteristics of the ionic media by using a solution dependent expression for the activity coefficient of the species involved in the equilibrium reactions. The other method uses an extended Debye Hückel expression where activity coefficients depend only on ionic charge and ionic strength, and it accounts for the solution particular properties by introducing ionic pairing between solution ions and species involved in the equilibrium reactions. The accurate determinations of logβ and logβ^0^ values remain challenging and reported values can vary significantly^29,30^. For example, the stability constant reported for [Zn(Cit)]^-^ in 0.1 mol dm^-3^ KNO_3_ differ by 1.36 log units^31,32^. A conditional stability constant can be adjusted for ionic strength and ion interactions by calculating activities of the species studied, i.e., a_i_ = γ_i_ [C_i_], where a is the activity of the species i, γ is the activity coefficient and C is the analytically determined concentration of species i. The Davies model has been used extensively in soil studies to calculate activity coefficients of electrolytes at fairly low ionic strengths^33^. The equation has no theoretical foundation but is found to work reasonably well up to 0.1 mol dm^−3^^33^. Another widely applied approach in metal–ligand coordination studies determines conditional stability constants at a number of points within the ionic strength range of interest and uses adjustable parameters to fit the Debye-Hückel model^22,26,27^. This approach has been termed as direct method or extended Debye Hückel (EDH) model^3,22,34^. The Debye-Hückel type model is expressed by the equation

1 $$\mathrm{log}{\upbeta }^{0}=\mathrm{log \upbeta }-0.51{\mathrm{z}}^{*}\frac{\surd \mathrm{I}}{1+1.5\surd \mathrm{I}}+f(I)$$

where $\left(I\right)$ is a linear function of ionic strength that can be formulated in different ways. The simplest expression for this term is $f\left(I\right)=CI$, with $C$ the only adjustable parameter. This simple choice is sufficient to explain the experimental data trend generally up to 1.0 mol dm^−3^.

To unravel the role of siderophores and other organic ligands during micronutrient transport in the rhizosphere is of particular interest to understand zinc efficiency mechanisms in rice genotypes^14,30,35–40^. The pH of the rice rhizosphere can vary by more than 2 pH units within 5 mm from the root surface and tends to remain within pH 4 and 8 irrespective of the mineralogical composition of the parent material^41–44^. The approximate ionic strength of soils where rice is grown is estimated to 0.007 mol dm^−3^^45,46^, however, due to sampling difficulties, it has not yet been possible to measure ionic strength gradients directly in the rice rhizosphere. Solute concentration profiles have been simulated for rice using root exudation rates^47^. As diffusion rates can be low, simulations suggest that solute concentration increases more than 30-times within 5 mm from the root surface^47^. Assuming the ionic strength gradient parallels the solute concentration gradient (which holds if anion/cation pairs are predominantly singularly charged), ionic strength is expected to range between 0.007 and 0.21 mol dm^−3^ in the rice rhizosphere. When testing the possibility of cooperative interactions between high and low affinity organic ligands during zinc transport in the rhizosphere, we need to demonstrate that solution conditions exist that enable the exchange of zinc ions from one ligand to the other, so-called ligand exchange points (LEPs)^2,3^. If we find pH and/or ionic strength windows at which one Zn(II)–ligand complex becomes more stable than another and these fall within the boundaries of the gradients expected in the rhizosphere, this would provide evidence for the model of cooperative interaction as it would imply that the two types of Zn(II)–ligand complex can dominate in different parts of the rhizosphere fulfilling different functions or the same function under different soil solution condition (see Fig. 1).

**Figure 2:**
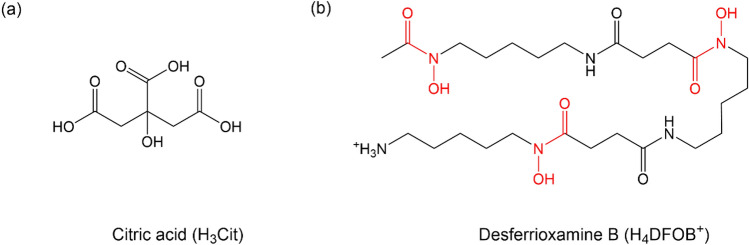
Chemical structure of the ligands under study. **(a)** Citric acid, shown as the fully protonated H_3_L ligand (previously reported pKa values extrapolated to zero ionic strength are 3.13, 4.76, 6.40^70^). **(b)** Desferrioxamine B (DFOB) with the three coordinating hydroxamic groups highlighted in red (each of this acts as a bidentate coordinating group). The structure is shown as the fully protonated [H_4_DFOB]^+^ structure—which includes the protonated amine (reported pK_a_ values at ionic strength of 1 mol dm^−3^ are 10.89, 9.61, 9.05 and 8.51^68^). At pH 7 or lower, DFOB is fully protonated, including the amine, i.e., it is present as [H_4_DFOB]^+^). The proposed coordination mode between Zn(II) and DFOB, based on solution studies rather than on structural data, is analogous to the one with iron, i.e., the zinc coordinates to the three hydroxamic acids, each of which is bidentate.

The aim of the present study was to determine the pH and ion strength dependent stability fields for Zn(II) complexes with citrate (low Zn affinity) and DFOB (high Zn affinity) and the location of the ligand exchange points using accurately determined intrinsic association constants. The determination of stability constants at different ionic strengths is critical to delineate the ionic strength stability field of the ligand metal complexes^33^. The coordination chemistry of Zn(II) with citrate and DFOB is well studied from solution and solid state studies (Fig. 2). This allows us to develop an accurate speciation model and to determine relevant association constants reliably and accurately, making DFOB and citrate an ideal model system. To this end, we first study the ionic strength dependence of Zn(II)–Cit and Zn(II)–DFOB stability constants using potentiometric titrations. We set up a complete chemical speciation model for both systems, determine conditional logβ values at multiple ionic strengths in NaCl and then fit the EDH model to the data to determine the intrinsic logβ^0^ using non-linear parametrisation. To our knowledge, this is the first time an accurate description of the ionic strength dependence of stability constants has been achieved for zinc in a system with a low and high affinity siderophores. We then determine the pH and ionic strength dependent LEPs for the exchange of zinc between citrate and DFOB in NaCl. We demonstrate that the predicted pH dependent LEPs fall within the gradient expected in the rice rhizosphere. Finally, we determine the pH and ionic strength dependent LEPs for the exchange of zinc between citrate and DFOB in soil solutions using the chemical composition of rice soil pore waters. We conclude that the concept of LEP and synergistic siderophore use is central to understand mobilisation and transport of zinc and other micronutrients in the rhizosphere and the reasons for multiple organic ligand production observed in soil and other environmental systems.

## Materials and method

### Chemicals

Zinc(II) solutions were prepared by dissolving the corresponding mass of ZnCl_2_ (99%, anhydrous, VWR) in water; the concentration was determined by complexometric titration against ethylenediaminetetraacetic acid (EDTA) standard solutions (Fisher Scientific). Standard hydrochloric acid (HCl) solutions were prepared from concentrated HCl (Sigma-Aldrich-Honeywell) and standardized with tris(hydroxymethyl)aminomethane (THAM) (Roche Diagnostics). CO_2_-free sodium hydroxide (NaOH) standard solutions were supplied by Fisher Scientific and were preserved from atmospheric CO_2_ by means of soda lime traps. Electrolyte solutions of sodium chloride (NaCl) were prepared from the pure salt (VWR). Citric acid monohydrate (VWR) and deferoxamine mesylate salt (Sigma-Aldrich) powders were used to prepare ligand solutions. Ultrapure water (R = 18 MΩ cm^−1^), grade A glassware, and analytical grade reagents were used throughout.

### Determination of stability constants

#### Potentiometric titrations

Potentiometric measurements were carried out at T = 298.1 ± 0.1 K in thermostatic cells. The setup consisted of a Metrohm model 888 Titrando apparatus controlled by Metrohm TiAMO 1.2 software equipped with a combined gel electrode (VWR model 662 1759)^34^. Estimated precision was ± 0.2 mV and ± 0.003 mL for the electromotive force and titrant volume readings, respectively. All the potentiometric titrations were carried out under magnetic stirring and bubbling purified pre-saturated N_2_ through the solution to exclude O_2_ and CO_2_.

Before studying the Zn(II)/ligand systems, the proton association constants of the ligands were determined at different ionic strengths (0.05 ≤ mol dm^−3^ ≤ 1.00) in NaCl solution. A 30 mL solution containing each ligand ([L] = 5 mmol dm^−3^), NaCl and HCl was titrated with standard NaOH solutions. For the Zn(II)/ligand systems, the titrant solutions consisted of different concentrations of ligand ([L] = 1 to 5 mmol dm^−3^), zinc ([Zn] = 0.5 to 1.5 mmol dm^−3^), a suitable amount of HCl and NaCl. All the measurements were carried out with equal or excess of the ligand with respect to the concentration of zinc. Solutions with Zn:L molar ratios of 1:1, 1:2, 1:5 and 1:10 were studied. Zinc and ligand concentrations were negligible compared to the background electrolyte. Calculations showed that ionic strength remained within 10% (v/v) of the targeted value throughout all titrations (see SI Fig. 1).

For each experiment, independent titrations of strong acid solutions with standard base were carried out under the same medium and ionic strength as the systems to be investigated, with the aim of determining the electrode potential (E^0^) using the GLEE software^48^. In this way, the pH scale used was the total scale, pH = −loga_H+_, where a_H+_ is the free proton activity. For each titration, approximately 80 to 100 data points were collected, and the equilibrium state during titrations was checked by confirming the time required to reach equilibrium.

#### Calculating stability constants from the potentiometric titration

The equilibrium reactions used in the speciation models for the two Zn(II)–ligand systems were chosen based on published solution and structural coordination studies^17,18,22,31,32,49–54^. We considered 1:1, 2:2 and 2:1 for Cit:Zn species and 1:1 for DFOB:Zn (see SI Table 1 for details). We did not consider the formation of ternary complexes since DFBO is an hexadentate complex, that unlikely would be outcompeted by citrate to form ternary complexes (e.g. [Zn(H_2_DFBO)(HCit)]^2−^). Such complex would imply that neither the DFOB nor the citrate are using their optimal denticity for coordination, i.e., hexadentate for DFOB and tridentate for citrate. In the proposed example above, DFOB would be tetradentate and citrate bidentate).

The software programs HySS and Hyperquad were used to determine experimental conditions for the titrations and to calculate stability constants from the potentiometric data set^55,56^. For each determination of a proton association constant, hydrolysis constant or Zn–L association constant at a given ion strength and L:Zn ratio, at least two different titrations were performed. The titration curves for each system were treated as a single set when refining stability constants. This meant that the refinement procedure was run on both curves at the same time to derive a single set of constants. During the fitting procedure, model results with sigma values above 5 were rejected. The error reported for the conditional stability constants is the standard deviation given in the Hyperquad output file^56^. Stability constants are defined as overall association constants (β). For a polyprotic acid (H_3_L) with three acidic sites, the β constants are expressed as:

2a $${\upbeta }_{{1}} = \, \left[ {{\text{HL}}} \right]^{{{2} - }} / \, \left[ {{\text{H}}^{ + } } \right]\left[ {{\text{L}}^{{{3} - }} } \right]$$

2b $${\upbeta }_{{2}} = \, \left[ {{\text{H}}_{{2}} {\text{L}}} \right]^{ - } / \, \left[ {{\text{H}}^{ + } } \right]^{{2}} \left[ {{\text{L}}^{{{3} - }} } \right]$$

2c $${\upbeta }_{{3}} = \, \left[ {{\text{H}}_{{3}} {\text{L}}} \right] \, / \left[ {{\text{H}}^{ + } } \right]^{{3}} \left[ {{\text{L}}^{{{3} - }} } \right]$$

Stepwise stability constants (K) are obtained from overall constants using the rule:

3 $${\upbeta }_{{2}} = {\text{ K}}_{{1}} {\text{K}}_{{2}}$$

The ionic strength model was developed by parametrising the EDH equation^57^. The logβ^0^ and C parameter were determined by optimisation using the solver function from Excel following a previously developed optimisation algorithm^58^. Monte Carlo simulations were subsequently used to estimate 95% confidence intervals on the parameters of ionic strength dependence following the procedure outlined elsewhere^59^. For each species, predicted stability constants were resampled using an inverse of the cumulative normal distribution function to give sets of simulated data from which the unknown parameters were optimized. The Davies equation is used to calculate the activity coefficient using

4 $$-\mathrm{log}{\gamma }_{i}=-\mathrm{A}{z}_{i}^{2}\left(\frac{\sqrt{\mathrm{I}}}{1+\sqrt{\mathrm{I}}}-0.3\mathrm{I}\right)$$

where γ_i_ is the activity coefficient, A is a dielectric constant of the solvent, z is the charge of the ion, and I is ion strength.

The formation constant of M_m_L_q_(OH)_n_, logβ, determined in NaCl (1:1 salt) of the ionic strength I, is related to the corresponding value at zero ionic strength, logβ^0^ by

5 $${\text{log}}\upbeta_{{}} = {\text{ log}}\upbeta^{0} + {\text{ mlog}}\gamma_{{\text{M}}} + {\text{ qlog}}\gamma_{{\text{L}}} + {\text{ bloga}}_{{{\text{H2O}}}} - {\text{ log}}\gamma \, - {\text{ nlog}}\gamma_{{\text{H}}}$$

### Thermodynamic and geochemical speciation modelling

Speciation calculations for the Zn(II)/citrate/DFOB systems in NaCl and in rice soil solutions were conducted using two computational speciation work packages, i.e. Hyperquad Simulation and Speciation computer software (HySS) and Visual MINTEQ^55,60,61^.

Speciation calculations with HySS used conditional and/or intrinsic stability constants determined experimentally during this study (Table 1). For all speciation calculations identifying the ligand exchange points, experiments were conducted using [Zn] = 10^–6^ mol dm^−3^ and [L] = 10^–5^ mol dm^−3^, which are the standard concentrations adopted studying the effectiveness of ligands including siderophores^2,21,62^. The sensitivity of the speciation calculations was determined running computations with high and low estimates for the stability constants. The maximum error was calculated using the difference between the results of these two different speciation calculations and was 5% for speciation calculations of solutions with 1 mol dm^−3^ and 0.5% for speciation calculations of solutions with 0.05 mol dm^−3^. We found no significance to the interpretations of the results; pH and ionic strength dependent LEPs shifted by < 0.01 pH units and < 0.001 mol dm^−3^, respectively.

**Table 1:** Ligand and proton association constants at different ionic strength (mol dm^−3^, in NaCl), T = 298.1 K.

Ligand	Equilibrium	0.05	0.15	0.3	0.5	1	log β_0_	C
Citrate	H^+^ + Cit^3-^ ⇌ HCit^2-^	5.69±0.01	5.44 ± 0.01	5.35 ± 0.01	5.26 ± 0.01	5.12 ± 0.01	6.198 ± .001	0.163 ± 0.001
	2H^+^ + Cit^3-^ ⇌ H^2^Cit^-^	10.18 ± 0.02	9.57 ± 0.02	9.45 ± 0.02	9.32 ± 0.02	9.13 ± 0.02	10.913 ± 0.005	0.254 ± 0.012
	3H^+^ + Cit^3-^ ⇌ H_3_Cit	13.00 ± 0.05	12.10 ± 0.03	11.94 ± 0.04	11.70 ± 0.03	11.53 ± 0.03	13.780 ± 0.006	0.142 ± 0.180
	Zn^2+^ + H^+^ + Cit^3−^ ⇌ [Zn(HCit)]	10.1 ± 0.1	8.52 ± 0.01	8.15 ± 0.02	7.83 ± 0.02	7.87 ± 0.02	10.640 ± 0.042	−0.147 ± 0.081
	Zn^2+^ + Cit^3−^ ⇌ ZnCit^−^	5.9 ± 0.1	4.885 ± 0.005	4.593 ± 0.006	4.310 ± 0.005	4.207 ± 0.005	6.583 ± 0.009	−0.057 ± 0.015
	Zn^2+^ + 2Cit^3−^ ⇌ [Zn(Cit)_2_]^4−^	nd	7.13 ± 0.04	6.85 ± 0.04	6.40 ± 0.06	6.88 ± 0.02	7.400 ± 0.07	0.284 ± 0.06
	2Zn^2+^ + 2Cit^3-^ + 2H_2_O ⇌ [Zn_2_(OH)_2_(Cit)_2_]^4-^ + 2H^+^	−1.8 ± 0.2	−2.61 ± 0.01	−2.81 ± 0.02	−3.09 ± 0.02	−2.51 ± 0.02	−1.142 ± 0.019	0.902 ± 0.033
	Zn^2+^ + Cit^3−^ + 3H_2_O ⇌ [Zn(OH)_3_(Cit)]^4-^ + 3H^+^	−21.9 ± 0.1	−22.46 ± 0.01	−22.92 ± 0.03	−22.63 ± 0.02	−23.05 ± 0.01	−21.601 ± 0.023	−0.248 ± 0.050
DFOB	H^+^ + DFOB^3-^ ⇌ HDFOB^2-^	11.07 ± 0.07	10.74 ± 0.04	10.36 ± .02	10.35 ± 0.02	10.14 ± 0.06	11.491 ± 0.002	0.169 ± 0.003
	2H^+^ + DFOB^3-^ ⇌ H_2_DFOB^-^	20.94 ± 0.09	20.25 ± 0.06	19.84 ± 0.04	19.77 ± 0.04	19.77 ± 0.08	21.530 ± 0.006	0.173 ± 0.011
	3H^+^ + DFOB^3-^ ⇌ H_3_DFOB	30.05 ± 0.12	29.10 ± 0.09	28.61 ± 0.07	28.60 ± 0.08	28.57 ± 0.12	30.691 ± 0.012	0.194 ± 0.028
	4H^+^ + DFOB^3-^ ⇌ H_4_DFOB^+^	38.83 ± 0.14	37.46 ± 0.13	37.00 ± 0.10	36.97 ± 0.16	36.92 ± 0.14	39.250 ± 0.023	−0.079 ± 0.043
	Zn^2+^ + DFOB^3-^ ⇌ [Zn(DFOB)]^-^	11.67 ± 0.06	9.91 ± 0.02	9.97 ± 0.04	9.39 ± 0.05	9.32 ± 0.04	12.027 ± 0.039	−0.486 ± 0.077
	Zn^2+^ + H^+^ + DFOB^3-^ ⇌ [Zn(HDFOB)]	21.55 ± 0.11	19.85 ± 0.04	19.85 ± 0.06	19.34 ± 0.09	19.38 ± 0.06	22.105 ± 0.026	−0.075 ± 0.053
	Zn^2+^ + 2H^+^ + DFOB^3-^ ⇌ [Zn(H_2_DFOB)]^+^	29.30 ± 0.13	27.81 ± 0.05	27.42 ± 0.08	27.19 ± 0.12	27.25 ± 0.08	29.849 ± 0.028	0.009 ± 0.051

The precisions  for the conditional (logβ) and intrinsic (logβ^0^) association constants are represented using experimental and model errors, respectively. The error for logβ is calculated from the titration fit using least quare optimisation within the Hyperquad program^55^. The error on the logβ^0^ is calculated using Monte Carlo simulations^59^.

*nd*  not determined.

Speciation calculations involving solutions with the chemical composition of pore waters from rice soil in Bangladesh, including dissolved organic carbon (DOC) used Visual MINTEQ with the NICA-Donnan model to account for metal-DOC interactions^61,63,64^. The concentration of DOC was 3.3 mmol dm^−3^ and based on this, the concentration of fulvic acids containing carboxylic and phenolic groups are assigned by Visual MINTEQ^65^. The concentration of zinc was set at 1 × 10^–8^ mol dm^−3^^66,67^. We modelled solutions with two different bicarbonate concentrations, i.e., 2 and 8 mmol dm^−3^, to account for the range of alkalinity found in calcareous rice-growing soils and to study the effect of bicarbonate complexation. The citrate and DFOB concentrations tested ranged between 1 and 50 µmol dm^−3^ and between 0.1 and 1 µmol dm^−3^ , respectively. The intrinsic stability constants (logβ^0^) for aqueous inorganic species, i.e., Zn(II)–chloride, Zn(II)–carbonate, Zn(II)–hydroxide, Zn(II)–phosphate were taken from the default database in Visual MINTEQ based on the National Institute of Standards and Technology (NIST) compilation^49^ and from a comprehensive data base compilation produced by Powell and co-workers^29^. The stability constants for citrate and DFOB species determined were added to/or amended in the database. The stability constants were adjusted when running the model at each new ionic strength so that the inbuilt Davies function used in Visual MINTEQ would set logβ at the desired value, i.e., the conditional value predicted by the appropriate EDH model.

Ligand exchange points (LEPs) were calculated by determining the pH or ionic strength condition at which the concentration of Zn(II)–DFOB and Zn(II)–Cit complexes were equal.

## Results and discussion

### Speciation models, conditional and intrinsic stability constants and EDH model parameters

**Figure 3:**
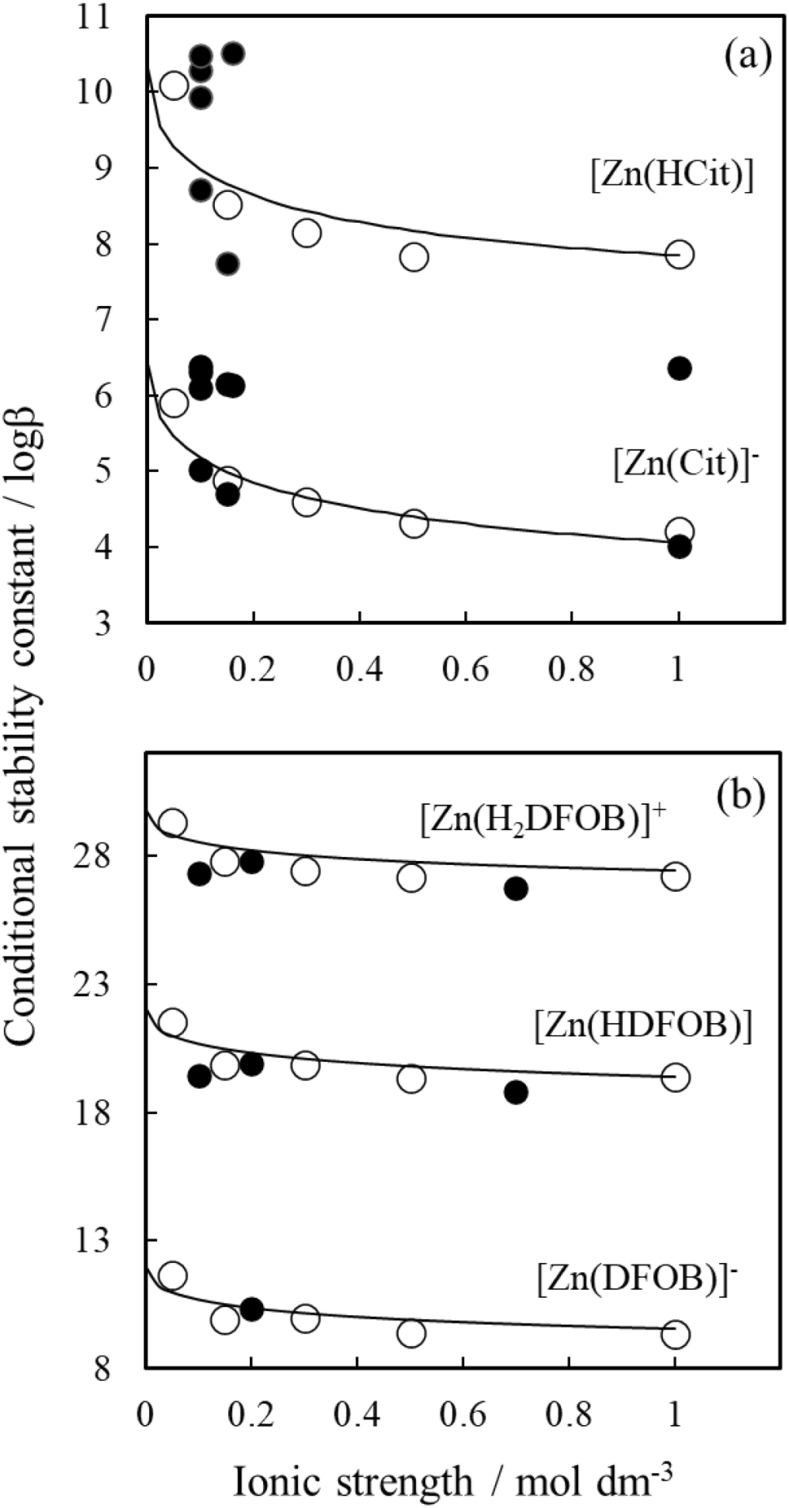
Experimental Zn(II)–ligand conditional stability constants (logβ) for **(a)** citrate and **(b)** DFOB at 0.05, 0.15, 0.3, 0.5 and 1 mol dm^−3^ in NaCl solution (open circles) determined using potentiometric titrations. For each species, the Extended Debye-Hückel (EDH) model has been parameterised using the experimental data (see Table 1 for C and logβ^0^) and the corresponding model is shown as a solid line. Literature data is included in the figure for comparison (closed circles) from Cigala et al. (2015, NaNO_3_ and NaCl), Capone et al. (1986, KNO_3_), Daniele et al. (1988, KNO_3_), Field et al. (1975, KNO_3_), Matsushima et al. (1963, NaCl) and Li et al. 1959, NaCl) for the Zn–H–Cit system and from Schijf et al. (2015, NaClO_4_), Farkas et al. (1997, KCl) and Hernlem et al. (1996, KNO_3_) for the Zn-H-DFOB system. Note the large variability reported for the Zn–Cit system at 0.1 and 0.15 mol dm^−3^. We find good agreement with the data published by Sammartano and co-workers^26,69^.

The complete set of analytical results for the Zn(II)/ligand systems, including conditional stability constants (logβ) for the formation of hydrolysed Zn(II)–ligand complexes, of zinc hydroxide complexes and of Zn(II)–ligand complexes as well as acidity constants for citrate and DFOB at different ionic strength in NaCl and T = 298.1 K are reported in Table 1 and SI Table 2. Also shown are the values for the optimised parameter C and the intrinsic association constants (logβ^0^). SI Table 1 lists all the reactions included in the speciation models used to fit the potentiometric titrations and SI Fig. 2 shows single crystal X-ray structures for some of the proposed structures including ZnH_2_Cit_2_, Zn_2_Cit_2_(H_2_O)_2_ and ZnCit_2_^2−^ taken from the Cambridge Crystallographic Data Base. Figure 3 displays the experimentally determined conditional Zn(II)–ligand stability constants and the corresponding EDH model from this study. Also shown are logb values from the literature for [Zn(HCit)] and [Zn(Cit)]^−^ for the Zn(II)/Cit system and [Zn(H_2_DFOB)]^+^, [Zn(HDFOB)] and [Zn(DFOB)]^−^ for the Zn(II)/DFOB system. Examples of titration curves and manually fitted models along with the speciation model considered and the experimental conditions are included in the supporting information (see SI Figs. 3 and 4). Only models that fitted the experimental data with sigma values below 5 were considered. Examples of Hyperquad files showing titrations and model fits for Zn(II)/Cit and Zn(II)/DFOB systems and of Excel calculation files for the application of the EDH model to the Zn(II)/DFOB experimental data set, including error calculation for C and logβ^0^ are uploaded to the Zenodo repository (https://doi.org/10.5281/zenodo.4548162). Errors reported for measured logβ and calculated (modelled) logβ^0^ and C values have no detectable effect on subsequent speciation calculations. The errors reported on C are slightly larger than in comparable studies^22^, however, a sensitivity analysis on the two Zn(II)–ligand species with the largest relative error on C found that logβ^0^ remains within its error range even when logβ^0^ was recalculated for the maximum and minimum possible C values. The stability constant we report for specific Zn(II)–L complexes at specific ion strengths are in line with literature reports (Fig. 3). For example, the logβ for the formation of [Zn(Cit)]^−^ in 0.15 mol dm^−3^ NaCl shows good agreement with the value reported by Cigala and co-workers in 0.15 mol dm^−3^ NaCl; 4.79 vs. 4.71^26^. We note, however, also significant variations within reported conditional logβ values as seen Fig. 3, with published values for the formation of [Zn(HCit)] and [Zn(Cit)]^−^ in different 1:1 electrolytes differing over two orders of magnitudes. This highlights the analytical challenges associated with accurate and precise logβ determinations of low affinity metal–ligand complexes, in low ion strength solutions^33^.

The final speciation scheme with the best statistical fits and with chemically sensible species are given in Table 1. From the eight Zn-Cit species initially considered (SI Table 1), the inclusion of five species resulted in model fits with sigma values below 5. For the Zn(II)/Cit system, the dominant species are [Zn(Cit)]^−^, [Zn(HCit)], and [Zn_2_(Cit)_2_(OH)_2_]^4−^. We report also the presence of a [Zn(Cit)(OH)_3_]^4−^ complex above pH 9 in significant amounts (> 20%) and we confirm the presence of [Zn(Cit)_2_]^4−^ if citrate is present in large excess^26,31^. The presence of [Zn(Cit)]^−^, [Zn(HCit)] and [Zn(Cit)_2_]^4−^ were confirmed in pH 6 solutions by mass spectrometry. To confirm the presence of [Zn(Cit)(OH)_3_]^4−^, further investigations are warranted. SI Fig. 5 shows the species distributions in the Zn(II)–Cit system with different Zn:L molar ratios (1:1, 1:2 and 1:10) and different concentrations (between 10^–6^ and 10^–3^ for Zn and 10^–5^ and 10^–3^ for citrate). We find that [Zn(Cit)]^−^ dominates (i.e., formation relative to total Zn is above 50%) between pH 5 and 7.5, [Zn_2_(Cit)_2_(OH)_2_]^4−^ dominates between pH 7.5 and 10 and [Zn(Cit)(OH)_3_]^4−^ dominates at pH values above 10. We find the formation of [Zn(Cit)_2_]^4−^ only at Zn:Cit molar ratio of 1:10 and [Zn] and [L] concentrations of 10^–4^ and 10^–3^ mol dm^−3^, respectively. The species [Zn(Cit)(OH)]^2−^ and Zn(Cit)(OH))_2_]^3−^ possibly form at higher pH but were excluded from the final model. We noted that for titrations of solutions with Zn:Cit molar ratios below 1:3, it was not possible to refine the stepwise stability constant (logK) for [Zn(Cit)_2_]^4−^ to within ± 0.09 log units, indicating that it is an unstable species that forms at negligible concentrations. The stability constants for zinc complexation with citrate decrease with increasing ionic strength. Table 1 shows that the most significant change is seen between 0.05 and 0.15 mol dm^−3^ NaCl, where there is approximately a 0.5 to 1.5 log unit change. In dilute solutions, stability constants are sensitive to small increases in ionic strength because changes in the effective concentration (activity) of ions are large.

For the Zn(II)–DFOB system, all the stability constants measured during this study are in good agreement with those reported in the literature^50,51,53^. For example, the stability constant we report for [Zn(HDFOB)] in 0.5 mol dm^−3^ NaCl is 19.34. This is within ~ 0.5 log units of the stability constant reported by Schijf and co-workers in 0.7 mol dm^−3^ NaClO_4_ solutions^53^. The speciation scheme we report differs slightly from that predicted by Schijf based on a three-step model. Our model does not include the bidentate species [Zn(H_3_DFOB)]^2+^, the weakest and least stable Zn(II)–DFOB species. In Table 1, we report stability constants for hexadentate [Zn(DFOB)]^−^ and [Zn(HDFOB)] and tetradentate [Zn(H_2_DFOB)]^+^. We observe that as the denticity of the complex increases, so does the strength of the stability constant. The stepwise stability constant (K) differs by approximately 2 log units between the formation of the three different DFOB:Zn:H species (7.75, 9.88, 11.67, see Table 1). DFOB complexation of Zn(II) shows the same pattern of ionic strength dependence as citrate, with the greatest decrease of logβ occurring between 0.05 and 0.15 mol dm^−3^ NaCl, the region of most importance to the rhizosphere.

The absolute decrease in [ZnL] and [Zn(HL)] stability constants between 0.05 and 0.15 mol dm^−3^ is approximately equal for citrate and DFOB species, average 1.58 vs. 1.73, respectively. This is explained by the effect of ionic strength primarily depending on the charge of the ions involved and free citrate and DFOB having the same electrostatic charge (−3). The ionic strength dependent parameter C shows no systematic change for neither citrate nor DFOB species. The good agreement between literature^50–52,54,68–70^ and our speciation models as well as the conditional logβ and pK_a_ values validates the use of a single analytical method for the determination of the LEP. We note that the proposed formation of the trihydroxy Zn(II) citrate complex at pH above 10, needs to be investigated in greater detail using supplementary techniques. However, the formation of this species is not relevant for the pH range of interest in our study. As discussed below the main prevailing species in solution are those of 1:1:0 and 2:2:−2 stoichiometry for Zn:Cit:H.

**Figure 4:**
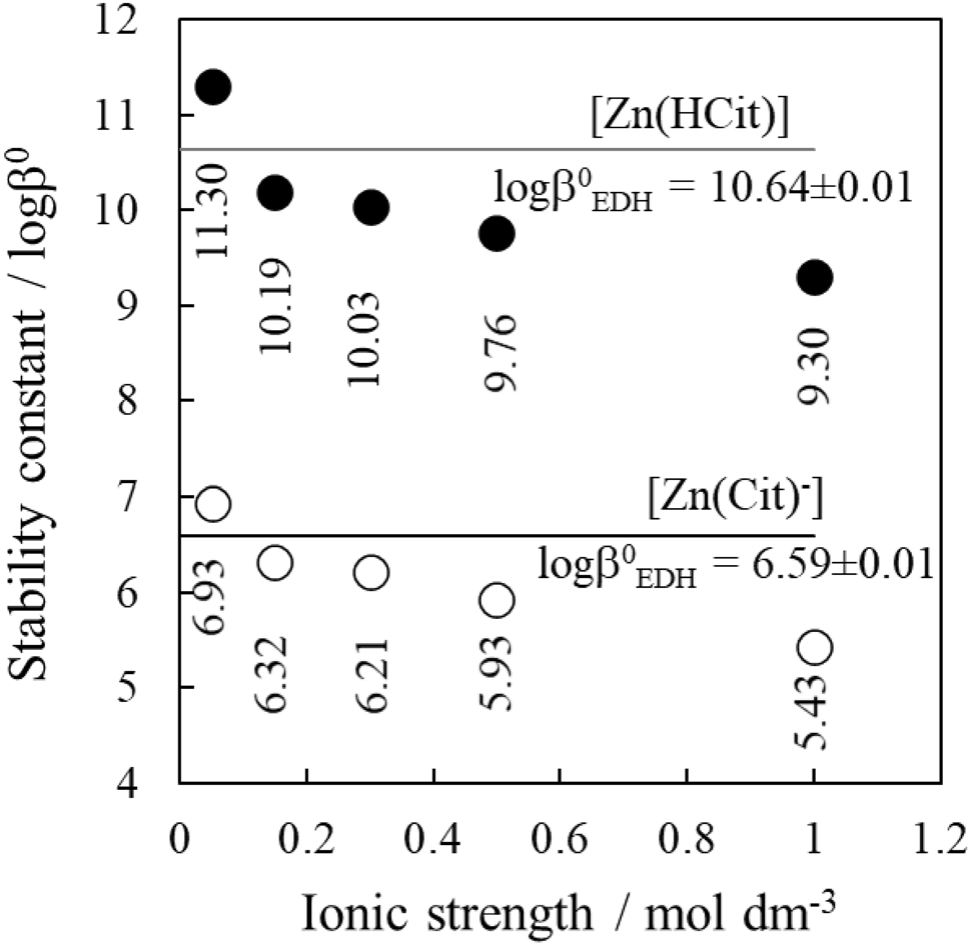
Intrinsic stability constants (logβ^0^) for the formation of [Zn(Cit)]^−^ and [Zn(HCit)] species calculated using the Davies equation and logβ values determined at different ionic strengths are shown as circles (open for [Zn(Cit)]^–^ and closed for [Zn(HCit)]). The intrinsic stability constants for the same two species determined by fitting the parameterised Extended Debye-Hückel (EDH) equation to the full ionic strength dataset are shown as solid lines. The error for the intrinsic stability constants is reported in SI Table 2 and differences are statistically significant (p < 0.05).

Figure 4 shows intrinsic stability constants for the formation of [Zn(Cit)]^−^ and [Zn(HCit)] determined (i) using the Davies equation and the conditional association constants determined at different ionic strengths and (ii) fitting the parameterised EDH equation to the full ionic strength dataset. We find statistically significant (p < 0.05) differences for the various logβ^0^, emphasizing that the method used for activity corrections is critical when studying the small changes in speciation happening during geochemical processes in the rhizosphere^3,30,34^. Intrinsic association constants seem over predicted below 0.1 mol dm^−3^ and underpredicted above 0.1 mol dm^−3^. The Excel file containing the calculations using Davies calculations is available on the Zenodo repository, https://doi.org/10.5281/zenodo.4548162.

**Figure 5:**
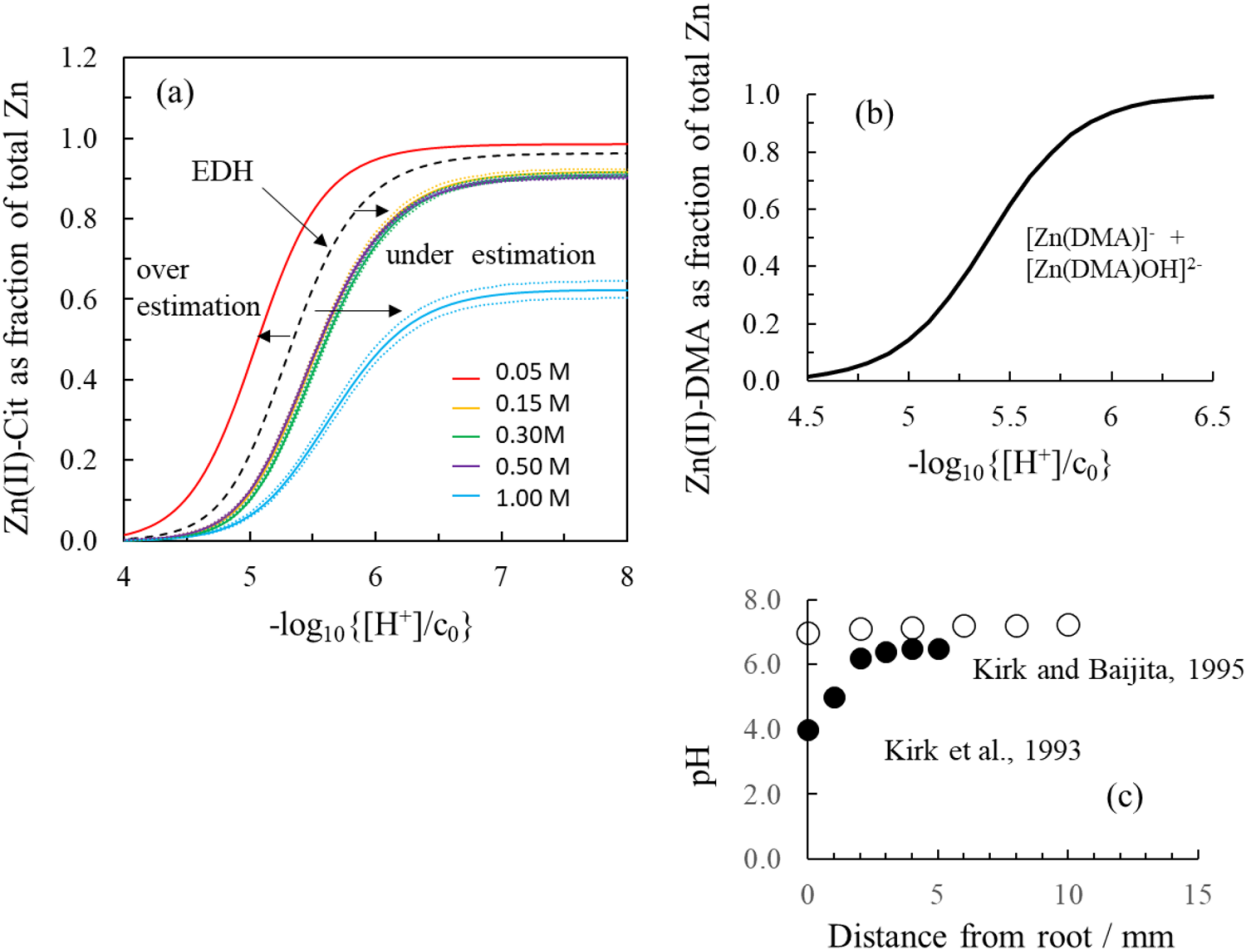
**(a)** Fraction of complexed zinc in a system with [Zn] = 10^–6^ mol dm^−3^ and [citrate] = 10^–5^ mol dm^−3^ modelled at infinite dilution using intrinsic stability constants determined (i) by fitting the parameterised version of the Extended Debye-Hückel equation to the full set of conditional stability constant data (ii–vi) by using the Davies equation to calculate activity coefficients and adjusting the citrate stability constants separately at 0.05, 0.15, 0.30, 0.50, and 1.00 mol dm^−3^. The dashed lines show the error on the respective curves. The error was calculated by re-running the analysis, starting with high and low estimates for the experimental stability constants. For the EDH and 0.05 mol dm^−3^ Davies corrected models, the error is too small to display. **(b)** Formation of Zn-DMA complexes in the pH range 2 and 8 calculated using logβ values from Weiss and co-workers^30^ with no corrections made for ionic strength dependent changes in formation constants. Note the similar range where Zn is complexed and the sigmoidal curve shape. **(c)** pH profiles between root wall and bulk solution measured in flooded rice fields^40,42^. pH at the root/soil interface can be acidic (pH  4), leading to the release of Zn from the complex.

Figure 5 shows the fraction of complexed zinc in a Zn(II)/Cit system modelled at infinite dilution using intrinsic stability constants determined (i) by fitting the EDH equation to the full citrate stability constant dataset or (ii-vi) using the Davies equation to calculate activity coefficients and adjust the citrate stability constants separately at 0.05, 0.15, 0.30, 0.50, and 1.00 mol dm^−3^. At pH 5.5, the 0.05 mol dm^−3^ Davies-based intrinsic speciation model overpredicts the fraction of complexed zinc by approximately 20% compared to the EDH-based intrinsic ion strength model. At the same pH value, the 0.15, 0.3, 0.5, and 1 mol dm^−3^ Davies-based intrinsic speciation models underpredict the fraction of complexed zinc by 18, 21, 20, and 38%, respectively, compared to the EDH-based intrinsic speciation model. These results demonstrate the inconsistencies in speciation calculations that arise when the same geochemical model is run using different sets of stability constants derived by applying the same method (e.g., Davies equation) to different sets of ionic strength data; even when the different sets of ionic strength data are within the activity model’s ionic strength range of applicability, i.e. for Davies equation < 0.5 mol dm^−3^ and from the same study^3,30,34^. This is a critical observation for  the pH range close to the root. A significant improvement in the accuracy of geochemical speciation calculations is achieved by adopting an empirical method with adjustable parameter when studying the ionic strength dependence of stability constants, rather than using an indirect method, as is widely practised as shown before^3,34^.

### pH and ionic strength dependent ligand exchange points (LEP) for between Zn(II)–citrate and Zn(II)–DFOB and stability windows for Zn(II)–ligand complexes

**Figure 6:**
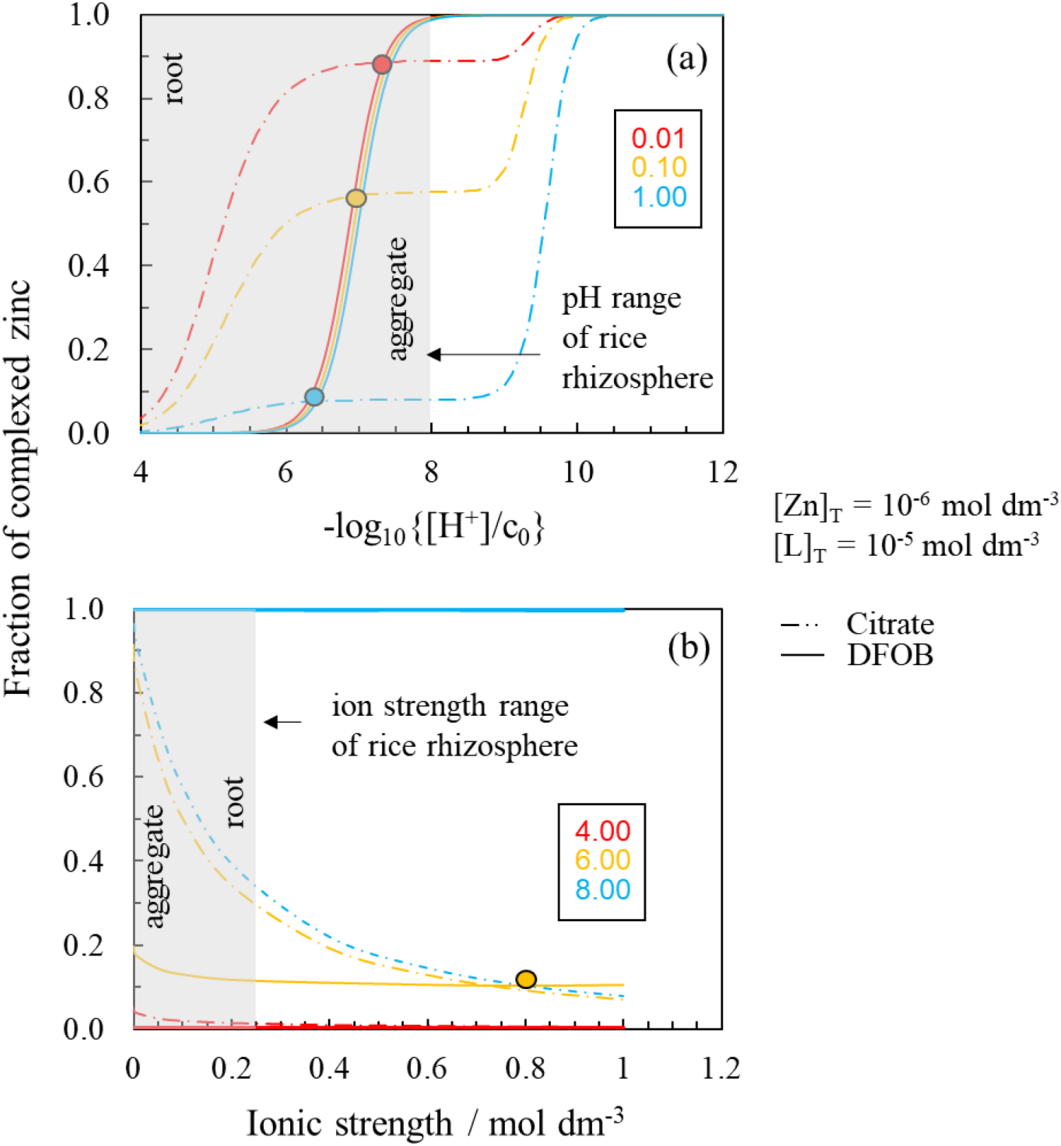
Fraction of complexed zinc in Zn(II)/Cit and Zn(II)/DFOB systems in NaCl solutions ([Zn] = 10^–6^ mol dm^−3^ and [L] = 10^–5^ mol dm^−3^) as a function of **(a)** pH and **(b)** ionic strength. The shaded grey areas show the rhizosphere-relevant pH and ionic strength windows along with the root and aggregate interfaces (see Fig. 1). The raw data for these plots is supplied in the supporting information (SI Tables 3 and 4).

The ionic strength dependence models described above were subsequently applied to investigate the geochemical stability window of the Zn(II)/ligand systems of interest and to determine the point of ligand exchange, i.e., where the fraction of complexed Zn(II)–Cit over fraction of complexed Zn(II)–DFOB is at unity. Figure 6 shows the fraction of complexed zinc in Zn(II)/Cit and Zn(II)/DFOB systems as a function of (a) pH and (b) ionic strength in NaCl solutions. The raw data for these plots are supplied in the supporting information (SI Table 4 and 5). We also show the range of I and pH values characteristically found between the root and soil aggregate interfaces in rice soils.

#### pH stability of Zn(II)–citrate and Zn(II)–DFOB complexes

For both the Zn(II)/citrate and Zn(II)/DFOB systems, the fraction of complexed zinc increases with pH as shown in Fig. 6.

For all the ionic strengths examined, Zn(II)–Cit complexes begin forming at approximately pH 3 (see SI Fig. 5 showing the species distribution for Zn(II)–Cit at different Zn:L molar ratios, ranging from 1:1 to 1:10 at 0.15 mol dm^−3^ NaCl solutions and different Zn and L concentrations). Once the citrate begins to bind to Zn, it takes between 6 to 7 pH units to reach total zinc complexation in the Zn(II)/Cit system. The fraction of zinc complexed by citrate increases fastest with pH in the lower ionic strength solution. At pH 6 in a 0.01 mol dm^−3^ NaCl solution, the fraction of zinc complexed by the citrate is 0.81 (SI Table 3). This is compared to just 0.07 at the same pH in a 1 mol dm^−3^ NaCl solution. The formation of Zn(II)–Cit complexes does not increase continuously with pH, there is a 2 to 3 pH unit plateau in the pH complexation curves for the Zn(II)/citrate system. Speciation diagrams for the Zn(II)/malate and Zn(II)/tartrate systems show similar trends to the Zn(II)/Cit pH complexation curves observed in this study; formation of Zn(II)–malate/tartrate complexes above 10% fraction of total zinc occurs at around pH 2 to 3 and there is then a plateau/only a small increase in the formation of Zn(II)–malate/tartrate complexes between pH 5 and 7^26^.

In the Zn(II)/DFOB system, ionic strength has a negligible effect on the pH complexation curves. In all solutions, complexation of zinc begins at pH 5.5 and total zinc complexation is reached within 3 pH units; no free zinc remains in the Zn(II)/DFOB systems above pH 8. The pH complexation curves for the Zn(II)/DFOB system are sigmoidal and do not contain a plateau (Fig. 6). Speciation diagrams for the Zn(II)/deoxymugineic acid (DMA), a rice produced siderophore^30^, system show a very similar pattern to the Zn(II)/DFOB pH dependent complexation curves in this study, suggesting this is a typical behaviour of high affinity siderophores. Significant concentrations of Zn(II)–DMA complexes begin forming at around pH 5 and total complexation of zinc is completed within 1.5 pH units^30^.

The pH at which Zn(II)–DFOB complexes become more abundant than Zn(II)–Cit complexes, i.e., the fraction of complexed zinc in the Zn(II)/DFOB system becomes greater than the fraction of complexed zinc in the Zn(II)/Cit system, depends strongly on ionic strength. As ionic strength increases, the pH dependent LEP becomes more acidic. In the 0.01, 0.1, and 1 mol dm^−3^ solutions, the pH dependent LEP is at pH 7.4, 7.1, and 6.5, respectively. This suggests that the thermodynamic favourability of the reaction for the exchange of zinc between citrate and DFOB increases with ionic strength.

For all ionic strengths tested, the predicted pH of the LEPs (pH 6.5 to 7.4) falls within the pH gradients expected in rhizospheres, which in the case of rice ranges between pH 4.0 and 8.0^40,42^. This suggests that pH gradients make it possible for Zn(II)–Cit and Zn(II)–DFOB complexes to dominate in different parts of the rice rhizosphere and, therefore, for the ligands to function synergistically. At the root interface, if pH is below 5.5 as previously suggested, neither Zn(II)–Cit nor Zn(II)–DFOB complexes are stable, excluding the possibility of an uptake of the Zn–ligand complex. It suggests that the role of the organic ligands in the uptake of metals and Zn is in increasing the leaching from soil aggregates and not in the actual uptake. Pairing of organic ligands with low and high affinity for Fe has been demonstrated by abiotic studies showing that a combination of siderophores and oxalate enhances Fe dissolution^19^.

#### Ionic strength stability for Zn(II)–Cit and Zn(II)–DFOB complexes

As ionic strength increases, the stability of the Zn(II)–Cit complexes decreases. Between 0 and 1 mol dm^−3^ ion strength, the fraction of Zn(II) complexed by citrate decreases by 0.05, 0.84, and 0.89 at pH 4, 6, and 8, respectively; in all instances this represents a relative reduction in ligand binding efficiency of approximately 92%. The Zn(II)–Cit ionic strength complexation curves initially descend sharply, two-thirds of the reduction in binding efficiency occurs before 0.2 mol dm^−3^, the critical range relevant to rhizosphere gradients. For Zn(II)–DFOB complexes, ionic strength only influences the stability at pH 6. At pH 4, no Zn(II)–DFOB complexes are stable and at pH 8 Zn(II) is fully complexed by the siderophore at all ionic strength investigated. At pH 6, between 0 to 1 mol dm^−3^, the decrease in fraction of complexed Zn(II) is 0.09 in the Zn(II)/DFOB system. This represents a relative reduction in ligand binding efficiency of 45%. Hence, the ionic strength controls more the stability of Zn(II)–Cit complexes than the stability of Zn(II)–DFOB complexes; it is larger (relative reduction in ligand binding efficiency 92% vs. 45%) and it is relevant over a wider pH range.

The ionic strength for the LEP at pH 6 is approximately 0.7 mol dm^−3^ (Fig. 6). At pH 4, the citrate remains dominant over the DFOB up to 1 mol dm^−3^ and at pH 8, the siderophore is already dominant over the citrate at 0 mol dm^−3^. This suggests that ionic strength controlled LEP occurs at a lower ionic strength as pH increases and consequently the thermodynamic favourability of the reaction for the exchange of zinc between citrate and DFOB increases with pH. Between pH 6 and 8, the predicted LEP is at an ionic strength smaller than 0.7 mol dm^−3^. Between pH 7 and 7.5, the predicted LEP ranges from ion strengths of between 0.01 and 0.1 mol dm^−3^, which overlaps with the estimated ionic strength gradient expected in the rhizosphere, in the case of rice ranging between 0.01 to 0.30 mol dm^−3^. Hence, our calculations suggest that when the pH of the rhizosphere is circumneutral, ionic strength gradients make it possible for Zn(II)–Cit and Zn(II)–DFOB complexes to dominate in different parts of the rhizosphere and, therefore, for the ligands to function synergistically.

### Geochemical speciation calculations of the Zn/Cit/DFOB system in rice soil

**Figure 7:**
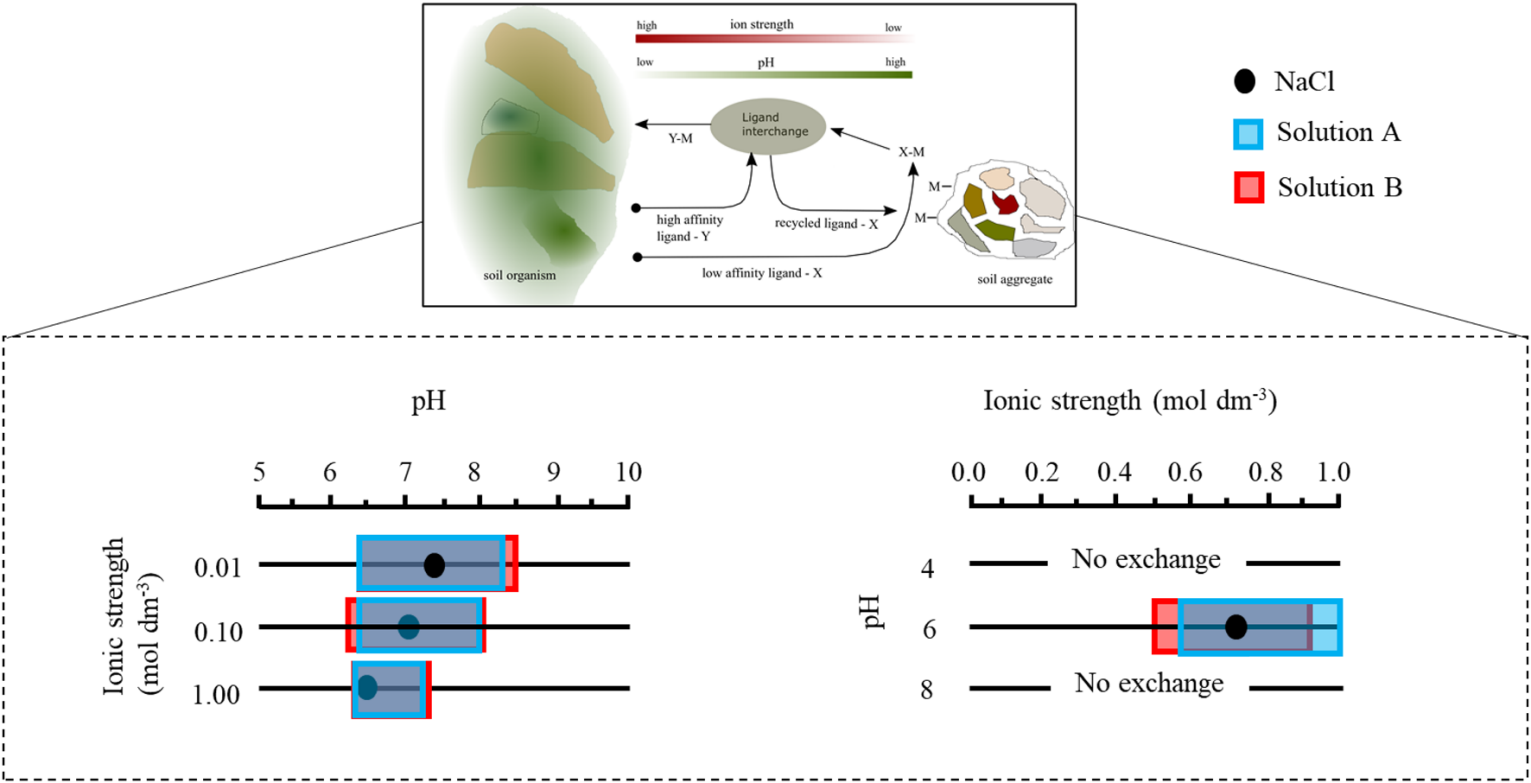
Calculated LEPs for the exchange of zinc between low affinity (citrate) and high affinity (DFOB) ligands in the presence of DOC (40 mg/l), Ca (64 mg dm^−3^), K (4.8 mg dm^−3^), Mg (22 mg dm^−3^), P 0.8 (mg dm^−3^), Si (8.7 mg dm^−3^), Co (2.2 μg dm^−3^), Ni (15.3 μg dm^−3^), Cu (11.1 μg dm^−3^), Se (2.5 μg dm^−3^), Cd (0.2 μg dm^−3^), Pb (1 μg dm^−3^), Fe (2.1 μg dm^−3^), Mn (3.2 μg dm^−3^), Na (0.1 mol dm^−3^) and Cl (0.1 mol dm^−3^). Concentration of Zn (10 nmol dm^−3^) and of other elements and DOC were based on published composition of pore water solutions of rice-growing soils^40,45,71^. Two model solutions were differentiated by the concentration of bicarbonate ions, i.e., solution A with 2 mmol dm^−3^ HCO_3_^−^ and solution B with 8 mmol dm^−3^ HCO_3_^−^. The predicted LEPs from the experiments in NaCl solutions with [Zn] = 10^–6^ mol dm^−3^ and [L] = 10^–5^ mol dm^−3^ (see Fig. 6 and Table 2) are included for comparison. The LEPs calculated for solution A and B are reported as ranges because different sets of citrates (1 and 50 µmol dm^−3^) and DFOB (0.1 and 1 µmol dm^−3^) concentrations were analysed.

Figure 7 and Table 2 show calculated LEPs for the exchange of zinc between citrate and DFOB in solutions with the chemical composition similar to that of rice soil porewater^44,45,71^. This modelling experiment aimed to assess the effect of competitive complexation by metals, dissolved organic carbon (DOC), and bicarbonate ions on the positions of the LEP and gaining insights into the importance of cooperative ligand interactions and of siderophores during zinc transport in rice soils at relevant metal and ligand concentrations as postulated previously^30,72^. The concentration of DOC used was 40 mg dm^−3^. The predicted LEP from the analysis in NaCl using standard zinc and ligand (for citrate and DFOB) concentrations are included for comparison. The LEPs are reported as ranges because different sets of citrate (1 to 50 µmol dm^−3^) and DFOB (0.1 to 1 µmol dm^−3^) concentrations were analysed in solutions with 2 mmol and 8 mol dm^−3^ HCO_3_^−^, denoted as solution A and B.

**Table 2:** Range of calculated LEPs in aqueous solutions with NaCl or with an elemental composition based on rice soil pore waters with different concentrations of bicarbonate, i.e., solution A with 2 mmol dm^−3^ and solution B with 8 mmol dm^−3^.

D		Ligand exchange point (LEP)		
		Solution A	Solution B	NaCl
Ion strength	0.01 mol dm^−3^	6.5–8.4	6.5–8.5	7.4
	0.10 mol dm^−3^	6.5–8.0	6.4–8.1	7.1
	1.00 mol dm^−3^	6.3–7.2	6.3–7.4	6.5
pH	4	nd	nd	nd
	6	0.58–1	0.48–0.92	0.73
	8	nd	nd	nd

Details of chemical composition are given in captions of Fig. 6.

We find that at all ionic strengths tested, the pH of the LEPs calculated in NaCl fall within the range calculated for the pH dependent LEPs in solutions with the chemical composition similar to that of rice soils. In line with the conclusions drawn above, it is not possible to calculate the ionic strength dependent LEPs in solution A or B at pH 4 or 8. This is because for the concentration and ion strength ranges tested, citrate is dominant at pH 4 and siderophore is dominant at pH 8. The ionic strength dependent LEP predicted in NaCl solutions at pH 6 falls within the range calculated for the ionic strength dependent LEPs in the model soil solutions. The pH dependent LEPs calculated in solution A and B at individual ionic strengths vary by between 0.9 and 2 pH units depending on the concentrations of citrate and DFOB used in the speciation calculations. The ionic strength depended LEPs vary by between 0.42 and 0.44 mol dm^−3^, depending on the concentrations of citrate and DFOB used in the speciation calculations. This evidence suggests that the LEPs in soil solutions are highly sensitive to ligand concentration ratios. Previous investigations have indeed highlighted the ligand concentration ratio as an important factor controlling the ligand-exchange process^73^. The size of the range calculated for the pH dependent LEPs decreases as ionic strength increases. This suggests that the ligand concentration ratio becomes less important in controlling the position of pH of the LEPs at higher ionic strength. The pH and ionic strength dependent LEPs in solution A and B are consistent i.e., the ranges broadly overlay one another. The two model solutions are differentiated by the concentration of bicarbonate ions, i.e., 2 and 8 mmol dm^−3^. The agreement would imply that the LEPs are not sensitive to bicarbonate concentration. This is in line with bicarbonate ions forming significant complexes with zinc only at higher pH^29^. Indeed, numerical modelling of Zn(II) speciation with Cl^–^, OH^–^, CO_3_^2–^, SO_4_^2–^, and PO_4_^3–^ using reliable stability (formation) constants showed that in acidic and weakly alkaline freshwater systems, in the absence of organic ligands, Zn(II) speciation is dominated by Zn^2+^^29^. The speciation of Zn(II) is dominated by ZnCO_3_ only at pH > 8.4. In seawater systems, the speciation at pH 8.2 is dominated by Zn^2+^ with ZnCl^+^, ZnCl_2_, Zn(CO_3_), and Zn(SO_4_) as minor species. Our results suggest furthermore that competition from DOC for zinc is not strong enough to shift the position of the LEPs. The role of DOC regarding possible complexation with zinc is expected to be minor given the structural properties of fulvic acids and the known low conditional stability constants for Zn(II)–DOC complexes, i.e., logK between ~ 3.8 and 4.2^74^.

SI Figs. 6 and 7 show the chemical speciation of Zn with DOC and environmental significant inorganic ligands (OH^−^, Cl^−^, CO_3_^2−^, SO_4_^2−^, PO_4_^3−^) in aqueous solutions with concentrations and elemental ratios found in rice soil pore waters. Within the pH range of importance to the rice rhizosphere, we find formation of Zn(HPO_4_), ZnDOC, ZnCl^+^, Zn(SO_4_) and Zn(CO_3_) only at very small abundances (below 20%).

## Conclusions

In this study, we have developed an accurate description of the ionic strength dependence of stability constants of Zn(II)–Cit and Zn(II)–DFOB complexes and we determined the stability windows of these complexes in synthetic (NaCl) and real world (rice soil) pore water solutions. Our study allows us to draw the following important conclusions.

1. The speciation model developed for citrate confirms the presence of [Zn(Cit)]^−^, [Zn(HCit)], [Zn_2_(Cit)_2_(OH)_2_]^4−^ and [Zn(Cit)_2_]^4−^ and proposes the presence of a [Zn(Cit)(OH)_3_]^4−^ complex above pH 9. The speciation model developed for DFOB verifies the existence of two hexadentate species, [Zn(DFOB)]^−^ and [Zn(HDFOB)], and one tetradentate species [Zn(H_2_DFOB)]^+^. We present accurate intrinsic and conditional association constants for the relevant Zn-ligand species.

2. Significant inaccuracies in speciation calculations arise when the same geochemical speciation model is run using intrinsic stability constants derived by applying the Davies equation to conditional affinity constants determined experimentally at ionic strength between 0 and 1 mol dm^−3^. When the Zn(II)/Cit system is modelled at infinite dilution using intrinsic stability constants determined using adjustable parameters and the EDH model, the accuracy of geochemical speciation calculations improves by at least 18% at pH 5.5, i.e. the pH of crucial importance with respect to zinc uptake into the root.

3. For all ionic strengths examined, Zn(II)–Cit complexes begin forming at approximately pH 3. Once the citrate begins binding to zinc, it takes between 6 to 7 pH units to reach total zinc complexation in the Zn(II)/citrate system. In the Zn(II)/DFOB system, ionic strength has a negligible effect on pH complexation curves. Complexation of zinc begins at pH 5.5 and total zinc complexation is reached within 3 pH units; no free zinc remains in the Zn(II)/DFOB systems above pH 8.

4. Variations in ionic strength affect the stability of Zn(II)–Cit complexes more than that of Zn(II)–DFOB complexes; it is larger (relative reduction in ligand binding efficiency is twofold) and it is relevant over a wider pH range. The pH range at which Zn(II)–DFOB complexes become more stable than Zn(II)–citrate complexes depends critically on the ionic strength. Ion strength gradients within the circumneutral pH of the rhizosphere make it possible for Zn(II)–Cit and Zn(II)–DFOB complexes to dominate in different parts of the rhizosphere and, therefore, for the ligands to function synergistically. This likely explains the previously observed synergistic effect of siderophores and weak organic acids enhancing Fe dissolution.

5. pH and ionic strength dependent LEPs for citrate and DFOB with zinc in NaCl solutions and in solutions based on the chemistry of rice soils fall within the pH and ionic strength gradients expected in the rhizosphere of rice soils. The LEPs in soil solutions are highly sensitive to ligand concentration ratios, in line with the idea that ligand concentration ratios play an control of ligand-exchange processes. The presence of DOC and inorganic ligands has no significant effect. 

6. Synergistic use of weak and strong binding organic ligands is an important mechanism for rice and other soil organisms to overcome micronutrient deficiencies. The synergistic use of organic ligands with high and low Zn affinity is possible due to pH and ion strength gradients found in the rhizospheres. Siderophores can have a distinct non-classical function to take up metals other than Fe and play an important role as zincophores in rice soils. In line with recent findings^3,24,30,34,36,43,72^, the present study supports the conclusion  that siderophores are central to access Zn in the soil.

## Supplementary Information

Supplementary Information.

## Data Availability

The datasets generated and/or analysed during the current study are available in the Zenodo repository, https://doi.org/10.5281/zenodo.4548162.
