# Supplementary material for: Synergistic use of siderophores and weak organic ligands during zinc transport in the rhizosphere controlled by pH and ion strength gradients
Source: Sci Rep. 2022 Apr 26;12:6774. doi: 10.1038/s41598-022-10493-5 (PMC9042811; doi:10.1038/s41598-022-10493-5)
Supplement: Supplementary file 1 — Supplementary Information. [file 41598_2022_10493_MOESM1_ESM.docx]

**Supplementary Information**

Synergistic use of siderophores and weak organic ligands during zinc transport in the rhizosphere controlled by pH and ion strength gradients

George H.R. Northover^1^, Yiru Mao^1^, Salvador Blasco^2^, Ramon Vilar^3^, Enrique Garcia-España^2^, Claudia Rocco^1,4^, Md Hanif^1,5^, and Dominik J. Weiss^1,6^

^1^Department of Earth Science and Engineering, Imperial College London, SW7 2AZ, United Kingdom

^2^Instituto de Ciencia Molecular (ICMol), University of Valencia, Paterna 46980, Spain

^3^Department of Chemistry, Imperial College London, W12 0BZ, United Kingdom

^4^MRC Centre for Molecular Bacteriology and Infection, Department of Life Sciences, Imperial College London, United Kingdom

^5^Soil, Water and Environment Department, Khulna University, Khulna, Bangladesh

^6^Department of Civil and Environmental Engineering, Princeton University, New Jersey 08540, United States of America

Author for correspondence: [d.weiss@imperial.ac.uk](mailto:d.weiss@imperial.ac.uk)

Author for correspondence: [d.weiss@imperial.ac.uk](mailto:d.weiss@imperial.ac.uk)

## SI Table 1

Reactions investigated during this study. The choice was based on published structural data from the Cambridge Crystallographic Data Centre and coordination chemistry studies in solution ^1-11^

|  |  |
| --- | --- |
| Number | Proposed reaction |
|  |  |
|  |  |
| 1 | Cit^3-^ + Zn^2+^ + 2H^+^ ⇌ [Zn(H_2_Cit)]^+^ |
| 2 | Cit^3-^ + Zn^2+^ + H^+^ ⇌ [Zn(HCit)] |
| 3 | Cit^3-^ + Zn^2+^ ⇌ [Zn(Cit)]^-^ |
| 4 | 2Cit^3-^ + Zn^2+^ + 2H^+^ ⇌ [Zn(HCit)_2_]^2-^ |
| 5 | 2Cit^3-^ + Zn^2+^ + H^+^ ⇌ [Zn(HCit)(Cit)]^3-^ |
| 6 | 2Cit^3-^ + Zn^2+^ ⇌ [Zn(Cit)_2_]^4-^ |
| 7 | 2Cit^3-^ + 2Zn^2+^ ⇌ [Zn_2_(Cit)_2_]^2-^ |
| 8 | 2Cit^3-^ + 2Zn^2+^ + 2OH^-^ ⇌ [Zn_2_(Cit)_2_(OH)_2_]^4-^ |
| 9 | Cit^3-^ + Zn^2+^ + OH^-^ ⇌ [Zn(Cit)(OH)]^2-^ |
| 10 | Cit^3-^ + Zn^2+^ + 2OH^-^ ⇌ [Zn(Cit)(OH)_2_]^3-^ |
| 11 | Cit^3-^ + Zn^2+^ + 3OH^-^ ⇌ [Zn(Cit)(OH)_3_]^4-^ |
|  |  |

## SI Table 2

Zn(II) hydrolysis constants (logβ) in aqueous solution with different NaCl concentrations (mol dm^-3^) at T = 298.1 K. Previously published values for [Zn(OH)_2_] are -17.8 ^12^, -17.82±0.08 ^13^, -16.9 ^14^ and -16.4 ^15^ and for [Zn(OH)_3_]^-^ -28.05±0.05 ^13^, -28.4 ^14^, -28.2±0.02 ^15^ and -28.1 ^12^. [Zn(OH)_2_] is the dominant species in the pH range relevant to the rhizosphere.

|  |  | |  | | |  | | |  | | |  | | |  |  |
| --- | --- | --- | --- | --- | --- | --- | --- | --- | --- | --- | --- | --- | --- | --- | --- | --- |
|  | |  | |  | | |  | | |  | | |  | | |  |
| Equilibrium | | 0.05 | | 0.15 | | | 0.3 | | | 0.5 | | | 1 | | |  |
|  | |  | | |  | | |  | | |  | | |  | | |
|  | |  | | |  | | |  | | |  | | |  | | |
| Zn^2+^ + 2H_2_O = [Zn(OH)_2_] + 2H^+^ | | -15.40±0.06 | | | -15.76±0.01 | | | -15.77±0.01 | | | -15.93±0.01 | | | -16.05±0.01 | | |
| Zn^2+^ + 3H_2_O = [Zn(OH)_3_]^-^ + 3H^+^ | | -25.16±0.09 | | | -26.15±0.04 | | | -26.10±0.03 | | | -26.51±0.04 | | | -26.56±0.03 | | |
|  | |  | |  | | |  | | |  | | |  | | |  |

## SI Table 3

Intrinsic stability constants for the zinc(II)/citrate system at T = 298.1 K calculated applying (i) the Davis model (see equation 4 in text) using experimentally determined conditional logβ values at different ionic strength (mol dm^-3^, NaCl) and (ii) the parametrised EDH model (see equation 1 in text).

|  |  |  |  | logβ^o^  Davies model | |  |  |
| --- | --- | --- | --- | --- | --- | --- | --- |
|  | Equilibrium | logβ^o^  EDH model |  |  |  |  |  |
|  |  |  | 0.05 M | 0.15 M | 0.3 M | 0.5 M | 1 M |
|  |  |  |  |  |  |  |  |
|  |  |  |  |  |  |  |  |
| 1 | H^+^ + Cit^3-^ ⇌ HCit^2-^ | 6.20±0.01 | 6.20±0.01 | 6.16±0.01 | 6.16±0.01 | 6.07±0.01 | 5.73±0.01 |
| 2 | 2H^+^ + Cit^3-^ ⇌ H_2_Cit^-^ | 10.91±0.01 | 11.04±0.02 | 10.76±0.02 | 10.80±0.02 | 10.67±0.02 | 10.15±0.02 |
| 3 | 3H^+^ + Cit^3-^ ⇌ H_3_Cit | 13.78±0.01 | 14.03±0.05 | 13.53±0.03 | 13.56±0.04 | 13.32±0.03 | 12.75±0.03 |
| 4 | Zn^2+^ + Cit^3−^ ⇌ [Zn(Cit)]^−^ | 6.58±0.01 | 6.93±0.01 | 6.32±0.04 | 6.21±0.03 | 5.93±0.01 | 5.43±0.04 |
| 5 | Zn^2+^ + 2Cit^3−^ ⇌ [Zn(Cit)_2_] | 7.40±0.01 |  | 6.77±0.04 | 6.45±0.03 | 6.00±0.01 | 6.57±0.04 |
| 6 | Zn^2+^ + H^+^ + Cit^3−^ ⇌ [Zn(HCit)] | 10.64±0.05 | 11.30±0.03 | 10.19±0.12 | 10.03±0.08 | 9.76±0.03 | 9.30±0.12 |
| 7 | Zn^2+^ + Cit^3−^ + 3H_2_O ⇌ [Zn(OH)_3_(Cit)]^4-^ + 3H^+^ | -21.60±0.02 | -21.39±0.01 | -21.74±0.05 | -22.11±0.03 | -21.82±0.03 | -22.44±0.05 |
| 8 | 2Zn^2+^ + 2Cit^3-^ + 2H_2_O ⇌ [Zn_2_(OH)_2_(Cit)_2_]^4-^ + 2H^+^ | -1.14±0.01 | -1.19±0.02 | -2.11±0.08 | -2.27±0.06 | -2.08±0.02 | -2.57±0.06 |
|  |  |  |  |  |  |  |  |

## SI Table 4

Fraction of complexed zinc in the Zn(II)/citrate and Zn(II)/DFOB systems as a function of pH in NaCl electrolyte solution with I = 0.01, 0.1 and 1 mol dm^-3^. [Zn] = 10^-6^ mol dm^-3^ and [L] =10^-5^ mol dm^-3^ .

|  |  |  |  |  |  |  |  |  |  |  |  |  |  |  |  |  |  |
| --- | --- | --- | --- | --- | --- | --- | --- | --- | --- | --- | --- | --- | --- | --- | --- | --- | --- |
| Ion strength | Ligand | Relative abundance | | | | | | | | | | | | | | | |
|  |  | pH | | | | | | | | | | | | | | | |
|  |  | 4 | 4.4 | 4.8 | 5.2 | 5.6 | 6 | 6.4 | 6.8 | 7.2 | 7.6 | 8 | 8.4 | 8.8 | 9.2 | 9.6 | 10 |
|  |  |  |  |  |  |  |  |  |  |  |  |  |  |  |  |  |  |
|  |  |  |  |  |  |  |  |  |  |  |  |  |  |  |  |  |  |
| 0.01 mol dm^-3^ | Cit | 0.036 | 0.119 | 0.304 | 0.538 | 0.717 | 0.814 | 0.859 | 0.878 | 0.885 | 0.888 | 0.889 | 0.890 | 0.891 | 0.920 | 0.986 | 0.999 |
|  | DFOB | 0.000 | 0.000 | 0.000 | 0.001 | 0.004 | 0.021 | 0.113 | 0.425 | 0.813 | 0.963 | 0.994 | 0.999 | 1.000 | 1.000 | 1.000 | 1.000 |
| 0.10 mol dm^-3^ | Cit | 0.019 | 0.061 | 0.153 | 0.289 | 0.418 | 0.502 | 0.545 | 0.563 | 0.571 | 0.575 | 0.576 | 0.577 | 0.591 | 0.734 | 0.960 | 0.997 |
|  | DFOB | 0.000 | 0.000 | 0.000 | 0.000 | 0.003 | 0.016 | 0.088 | 0.359 | 0.767 | 0.952 | 0.992 | 0.999 | 1.000 | 1.000 | 1.000 | 1.000 |
| 1.00 mol dm^-3^ | Cit | 0.004 | 0.011 | 0.025 | 0.044 | 0.061 | 0.071 | 0.076 | 0.078 | 0.079 | 0.079 | 0.080 | 0.081 | 0.092 | 0.179 | 0.574 | 0.948 |
|  | DFOB | 0.000 | 0.000 | 0.000 | 0.000 | 0.002 | 0.013 | 0.071 | 0.305 | 0.718 | 0.938 | 0.989 | 0.998 | 1.000 | 1.000 | 1.000 | 1.000 |
|  |  |  |  |  |  |  |  |  |  |  |  |  |  |  |  |  |  |

## SI Table 5

Fraction of complexed zinc in the Zn(II)/citrate and Zn(II)/DFOB systems as a function of ion strength in NaCl electrolyte solution at pH 4, 6 and 8, covering the pH range found in the rhizosphere. [Zn] = 10^-6^ mol dm^-3^ and [L] =10^-5^ mol dm^-3^ .

|  |  |  |  |  |  |  |  |  |  |  |
| --- | --- | --- | --- | --- | --- | --- | --- | --- | --- | --- |
| pH | Ligand | Ionic strength / mol dm^-3^ | | | | | | | | |
|  |  | 0 | 0.005 | 0.05 | 0.1 | 0.2 | 0.4 | 0.6 | 0.8 | 1 |
|  |  |  |  |  |  |  |  |  |  |  |
|  |  |  |  |  |  |  |  |  |  |  |
| 4 | Cit | 0.052 | 0.040 | 0.025 | 0.019 | 0.014 | 0.009 | 0.007 | 0.005 | 0.004 |
|  | DFOB | 0.000 | 0.000 | 0.000 | 0.000 | 0.000 | 0.000 | 0.000 | 0.000 | 0.000 |
| 6 | Cit | 0.914 | 0.848 | 0.644 | 0.502 | 0.342 | 0.193 | 0.129 | 0.093 | 0.071 |
|  | DFOB | 0.195 | 0.175 | 0.144 | 0.130 | 0.118 | 0.111 | 0.106 | 0.104 | 0.106 |
| 8 | Cit | 0.965 | 0.917 | 0.727 | 0.576 | 0.393 | 0.220 | 0.146 | 0.104 | 0.080 |
|  | DFOB | 1.000 | 1.000 | 1.000 | 1.000 | 1.000 | 1.000 | 1.000 | 1.000 | 1.000 |
|  |  |  |  |  |  |  |  |  |  |  |

## SI Figure 1

Effect of titration on ion strength due to dilution/concentration of titration solution.


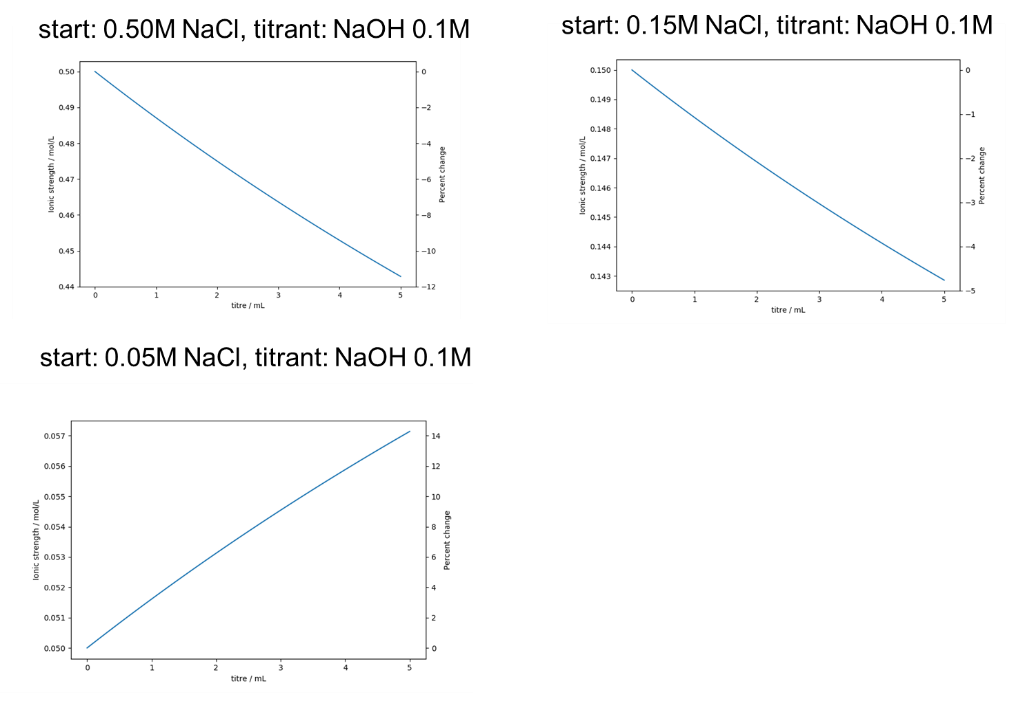


## SI Figure 2

Single-crystal X-ray structures for ZnH_2_L_2_ (a), Zn_2_L_2_(H_2_O)_2_ (b) and ZnL_2_^2-^ (c) taken from Cambridge Crystallographic Data Centre. Structures a and c just differ in the protonation of the carboxylate groups.


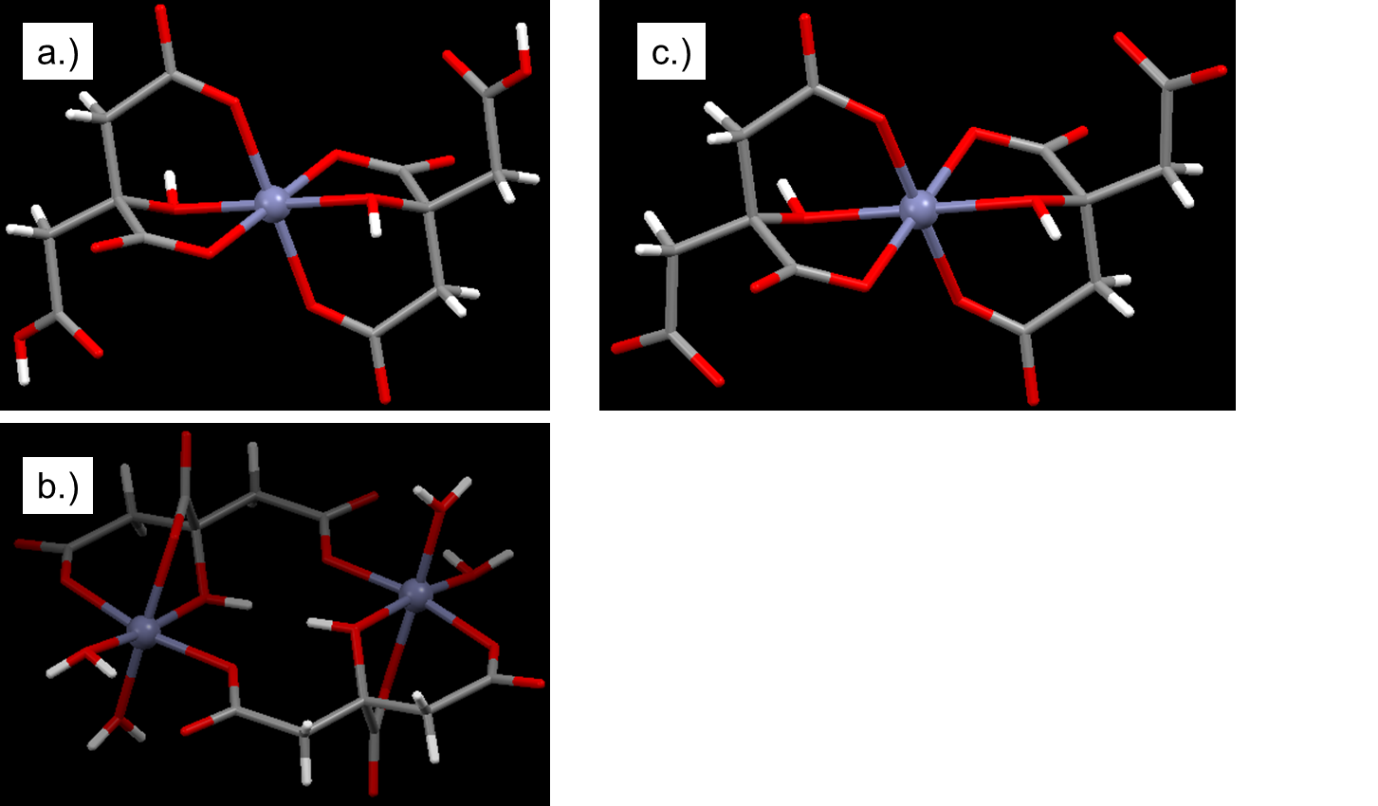


## SI Figure 3

Manual fitting of potentiometric data in Hyperquad for the Zn(II)/Cit system

## SI Figure 4

Manual fitting of potentiometric data in Hyperquad for the Zn(II)/DFOB system

## SI Figure 5

Species distribution of the Zn(II)/Cit system for

(a) Zn:L = 1:1, [Zn] = [L] = 0.001 mol dm^-3^,

(b) Zn:L = 1:2, [Zn] = 0.0005 mol dm^-3^ and [L] = 0.001 mol dm^-3^,

(c) Zn:L = 1:10, [Zn] = 0.0001 mol dm^-3^ and [L] = 0.001 mol dm^-3^ and

(d) Zn:L = 1:10, [Zn] = 1 μmol dm^-3^ and [L] = 10 μmol dm^-3^.


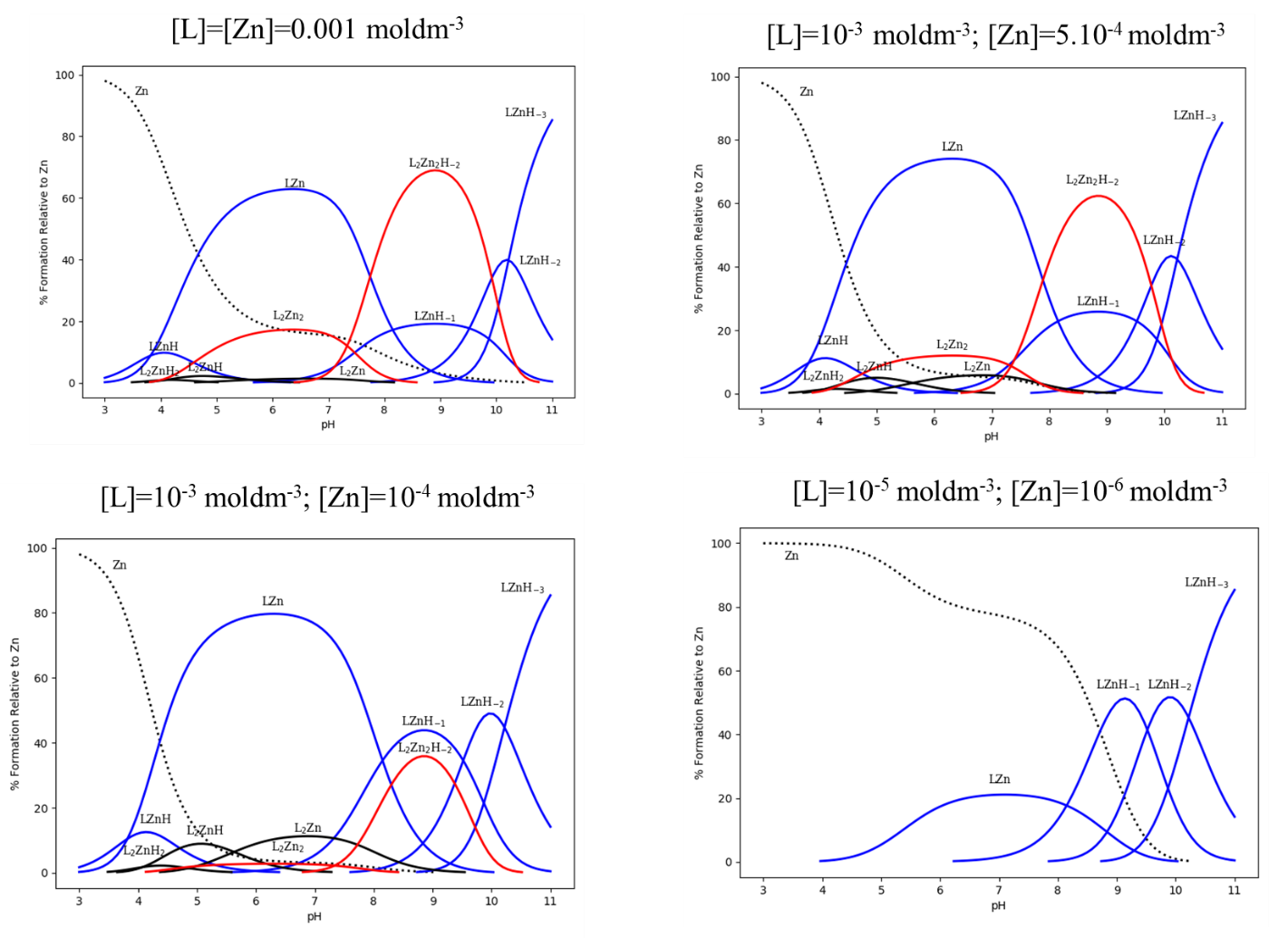


## SI Figure 6

Speciation diagram for the Zn^2+^ + H^+^ + Cl^-^ + CO_3_^2-^ + PO_4_^3–^ + DOC system with [Cl^-^] = 0.1 mol dm^–3^, [DOC] = 40 mg dm^–3^, [CO_3_^2-^] = 2 mmol dm^–3^, [PO_4_^3–^] = 20 mg dm^-3^ and [Zn] = 75 mmol dm^–3^. It was assumed that the system has a pe = 4, representative for rice soils at the beginning of the flooding cycle ^16^. The formation of Zn-DOC complexes is minimal.


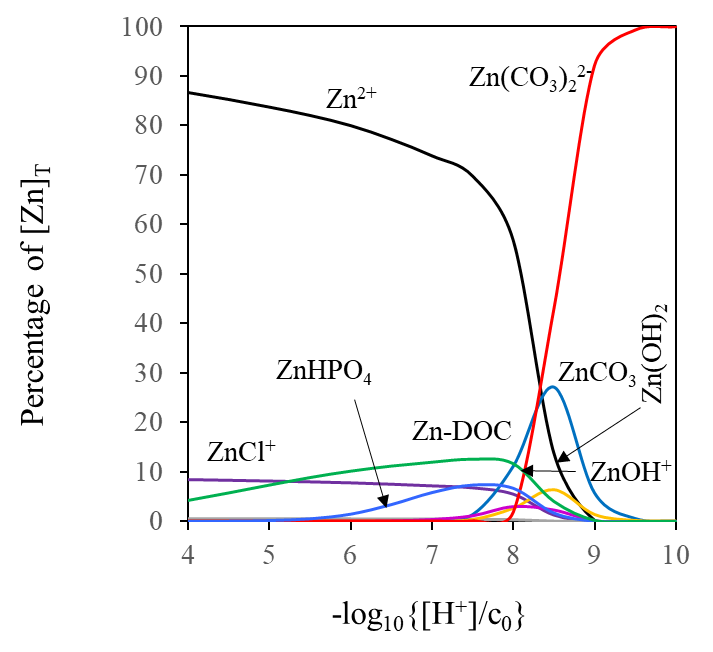


## SI Figure 7

Speciation diagram for the Zn^2+^ + H^+^ + Cl^-^ + CO_3_^2-^ + PO_4_^3–^ + SO_4_^2–^ system with [Cl^-^] = 0.23 mmol dm^–3^, [SO_4_^2–^] = 0.42 mmol dm^–3^, [PO_4_^3–^] = 0.7 μ mol dm^–3^, [CO_3_^2-^] = 8 mmol dm^–3^ and [Zn] = 1 nmol dm^–3^. All formation constants are taken from ^17^. PO_4_^3–^:Zn molar ratios are similar to these found in porewater of Bangladesh rice soils ^18^.


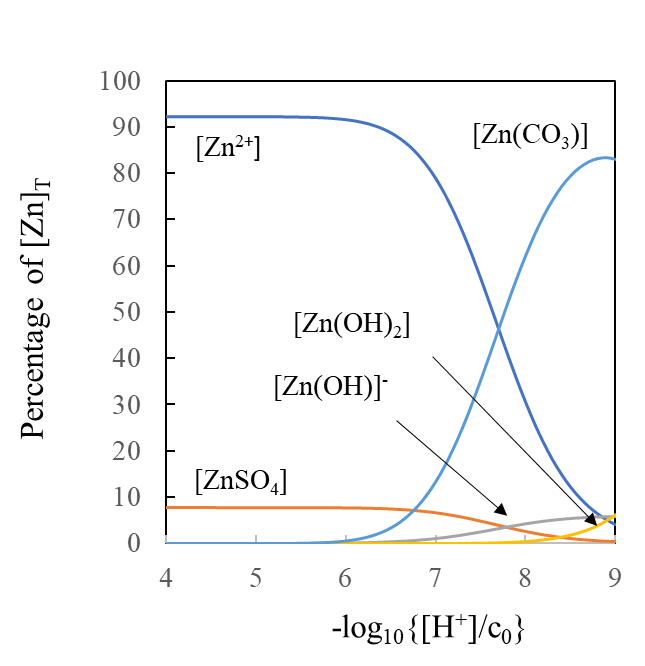


## References

1 Capone, S., De Robertis, A., De Stefano, C. & Sammartano, S. Formation and stability of zinc(II) and cadmium(II) citrate complexes in aqueous solution at various temperatures. *Talanta* **33**, 763-767, doi:10.1016/0039-9140(86)80184-9 (1986).

2 Daniele, P. G., Rigano, C. & Sammartano, S. Ionic strength dependence of formation constants-I. Protonation constants of organic and inorganic acids. *Talanta* **30**, 81-87, doi:10.1016/0039-9140(83)80023-X (1983).

3 Capone, S., De Robertis, A., De Stefano, C., Sammartano, S. & Scarcella, R. Ionic strength dependence of formation constants-X. Proton activity coefficients at various temperatures and ionic strengths and their use in the study of complex equilibria. *Talanta* **34**, 593-598, doi:10.1016/0039-9140(87)80198-4 (1987).

4 Cigala, R. M. *et al.* Quantitative study on the interaction of Sn2+and Zn2+with some phosphate ligands, in aqueous solution at different ionic strengths. *Journal of Molecular Liquids* **165**, 143-153, doi:10.1016/j.molliq.2011.11.002 (2012).

5 Li, N. C., Lindenbaum, A. & White, J. M. Some metal complexes of citric and tricarballylic acids. *Journal of Inorganic and Nuclear Chemistry* **12**, 122-128, doi:10.1016/0022-1902(59)80101-9 (1959).

6 Cigala, R. M. *et al.* Speciation of tin(II) in aqueous solution: Thermodynamic and spectroscopic study of simple and mixed hydroxocarboxylate complexes. *Monatshefte fur Chemie* **144**, 761-772, doi:10.1007/s00706-013-0961-3 (2013).

7 Guggenheim, E. A. & Turgeon, J. C. Specific interaction of ions. *Transactions of the Faraday Society* **51**, 747-761, doi:10.1039/TF9555100747 (1955).

8 Hernlem, B. J., Vane, L. M. & Sayles, G. D. Stability constants for complexes of the siderophore desferrioxamine b with selected heavy metal cations. *Inorganica Chimica Acta* **244**, 179-184, doi:10.1016/0020-1693(95)04780-8 (1996).

9 Deng, Y.-F. & Zhou, Z.-H. Synthesis and crystal structure of a zinc citrate complex [Zn(H2cit)(H2O)] n. *Journal of Coordination Chemistry* **62**, 1484-1491, doi:10.1080/00958970802596391 (2009).

10 Goldberg, R. N., Kishore, N. & Lennen, R. M. Thermodynamic Quantities for the Ionization Reactions of Buffers. *Journal of Physical and Chemical Reference Data* **31**, 231-370, doi:10.1063/1.1416902 (2002).

11 Cigala, R. M. *et al.* Zinc(II) complexes with hydroxocarboxylates and mixed metal species with tin(II) in different salts aqueous solutions at different ionic strengths: Formation, stability, and weak interactions with supporting electrolytes. *Monatshefte fur Chemie* **146**, 527-540, doi:10.1007/s00706-014-1394-3 (2015).

12 Smith, R. M. & Martell, A. E. Critical stability constants. *Inorganic Complexes* **4** (1976).

13 Powell, K. J. *et al.* Chemical speciation of environmentally significant metals with inorganic ligands. Part 5: The Zn2+ + OH-, Cl-, CO32-, SO42-, and PO43-systems (IUPAC Technical Report). *Pure and Applied Chemistry* **85**, 2249-2311, doi:10.1351/PAC-REP-13-06-03 (2013).

14 Baes, C. F. & Mesmer, R. E. *The hydrolysis of cations*. (John Wiley and Sons, 1976).

15 Zhang, Y. & Muhammed, M. Critical evaluation of thermodynamics of complex formation of metal ions in aqueous solutions - VI. Hydrolysis and hydroxo-complexes of Zn2+ at 298.15 K. *Hydrometallurgy* **60**, 215-236, doi:10.1016/S0304-386X(01)00148-7 (2001).

16 Fageria, N. K., Carvalho, G. D., Santos, A. B., Ferreira, E. P. B. & Knupp, A. M. Chemistry of Lowland Rice Soils and Nutrient Availability. *Communications in Soil Science and Plant Analysis* **42**, 1913-1933, doi:10.1080/00103624.2011.591467 (2011).

17 Powell, K. J., Brown, P. L., Byrne, R. H. & Gajda, T. Chemical Speciation of Environmentally Significant Metals. 15-19, doi:10.1351/pac200779050895 (2015).

18 Williams, P. N. *et al.* Organic Matter—Solid Phase Interactions Are Critical for Predicting Arsenic Release and Plant Uptake in Bangladesh Paddy Soils. *Environmental Science & Technology* **45**, 6080-6087, doi:10.1021/es2003765 (2011).
